# Benefits of multinomial processing tree models with discrete and continuous variables in memory research: an alternative modeling proposal to Juola et al. (2019)

**DOI:** 10.3758/s13421-023-01501-8

**Published:** 2024-01-04

**Authors:** Anahí Gutkin, Manuel Suero, Juan Botella, James F. Juola

**Affiliations:** 1 Department of Psychological Methods, Philipps-Universität Marburg, Marburg, Germany; 2 Department of Social Psychology and Methodology, Universidad Autónoma de Madrid, Madrid, Spain; 3 Department of Social Psychology, University of Kansas, Lawrence, Kansas USA

**Keywords:** Multinomial processing tree, Recognition memory models, Confidence ratings, Reaction time

## Abstract

Signal detection theory (SDT) and two-high threshold models (2HT) are often used to analyze accuracy data in recognition memory paradigms. However, when reaction times (RTs) and/or confidence levels (CLs) are also measured, they usually are analyzed separately or not at all as dependent variables (DVs). We propose a new approach to include these variables based on multinomial processing tree models for discrete and continuous variables (MPT-DC) with the aim to compare fits of SDT and 2HT models. Using Juola et al.’s (2019, *Memory & Cognition, 47*[4], 855–876) data we have found that including CLs and RTs reduces the standard errors of parameter estimates and accounts for interactions among accuracy, CLs, and RTs that classical versions of SDT and 2HT models do not. In addition, according to the simulations, there is an increase in the proportion of correct model selections when relevant DV are included. We highlight the methodological and substantive advantages of MPT-DC in the disentanglement of contributing processes in recognition memory.

Theories of cognitive architectures and processes can perhaps best be evaluated by deriving quantitative predictions that can be tested against data from behavioral experiments. Determining which mathematical models best describe human behavior is an interesting way to evaluate theories and a crucial step towards finding methods to answer our research questions. To model effectively, the types of variables involved (nominal, ordinal, interval, or ratio) and the roles they play in specific experimental paradigms (as dependent or independent variables, etc.) must be defined, and all variables that carry relevant information should be included in the model. In the present study, we will illustrate this point by focusing on recognition memory models.

In standard recognition-memory experiments, participants first study a list of items (usually words) and then try to identify items presented in a test phase as to whether they had been previously studied or not. Those words that had been previously studied are to be classified as “old” and nonstudied words are to be called “new.” By knowing the type of word presented for a test and the response of the participant, we can distinguish four categories of responses (see Table 1): previously studied (target) words result in a Hit (Hit: “old” response to an old item) or a Miss (Miss: “new” response to an old item), whereas new (distractor) words can result in a false alarm (FA: “old” response to a new item) or a correct rejection (CR: “new” response to a new item). In order to explain the experimental results obtained with this paradigm, two classes of mathematical models have been fitted: signal detection theory models (SDT; Ashby, 2014; Atkinson & Juola, 1973, 1974; Juola et al., 1971; Luce, 1959; Thurstone, 1927) and multinomial processing trees, MPT, which include the two-high-threshold (2HT) model (Batchelder & Riefer, 1990; Bröder & Schütz, 2009; Erdfelder et al., 2009; for a recent MPT tutorial, see Schmidt et al., 2023).

**Table 1:** Recognition paradigm categories

Presented Stimulus	Participant’s response	
	“Old”	“New”
Old	Hit	Miss
New	FA	CR

Although these models have proven useful because they were originally designed to include categorical variables, recognition experiments often also measure continuous variables such as response times (RTs), besides ordinal variables such as confidence levels (CLs). Traditional SDT and 2HT models make predictions about these variables, and also about speed–accuracy relationships (Atkinson & Juola, 1973; Hockley, 1982; Murdock & Dufty, 1972). However, when both categorical and quantitative variables are measured (e.g., by recording RT in a recognition task), they are usually analyzed separately, or one variable is just ignored. Such practices have several limitations.

First, there is a risk of interpreting only the significant effects of one variable (e.g., RT) without considering the other one (e.g., accuracy), which would be prone to the accumulation of Type I errors (Voss et al., 2013). Second, because of the well-known speed–accuracy trade-off effect, we cannot predict whether a participant will prioritize performing the task well or on performing it fast. The response strategy may vary among individuals, items, experimental designs, instructions, and conditions, and this will affect both RT and accuracy Tourangeau et al. (2000). Consequently, we cannot have an overall interpretation when ignoring one variable, and analyzing both measures separately can lead to incongruent results (Liesefeld, & Janczyk, 2019). Furthermore, when considering only one variable we lose information about the other and this can affect the accuracy of parameter estimates. Also, if individual differences in performance are distributed between the two metrics (e.g., RTs and accuracy), we might not detect significant effects in either of them. Thus, we could see a reduction in statistical power (Voss et al., 2013). Psychometry has addressed this issue by using hierarchical models based on item response theory (Molenaar et al., 2015; van der Linden, 2007), and it has demonstrated that if two dimensions are related, the estimates of one are improved when the other’s data are analyzed jointly. These psychometric models are applicable to many research areas because they are general measurement models and are not limited by any specific process models (De Boeck, & Jeon, 2019; Wagenmakers, 2009).

Thanks to recent developments in MPT models, many previous obstacles can be overcome, and quantitative and categorical variables can be modeled jointly (Heck & Erdfelder, 2016; Heck et al., 2018b; Klauer & Kellen; 2018; Schweickert & Zheng, 2019). MPT models for discrete and continuous variables (MPT-DC) are process models with a theoretical rationale that assumes that processes have intrinsic attributes (e.g., processing time), and modeling these variables can help to identify and measure these attributes and their theoretical sources. Therefore, MPT-DC have both methodological and theoretical benefits. These models could yield different conclusions than models that do not jointly analyze quantitative and categorical variables. Furthermore, they allow us to make new inferences about the structure and functioning of cognitive models, study the relationships between dependent variables including trade-offs and lead to a finer understanding of how individuals process information and respond accordingly.

Quantitative variables, such as RT (Heck & Erdfelder, 2016, 2020; Klauer & Kellen, 2018) and both RT and CLs jointly (Starns, 2021) have been included in 2HT models through MPT-DC models. Here we propose to include both RT and CLs jointly in SDT models by a solution based on reparametrizing SDT as multinomial models (estimating SDT models as multinomial models has previously been proposed by Singmann & Kellen, 2013). To the best of our knowledge, the extension of both MPT-DC and SDT models to RT and CL data has not yet been developed.

In short, MPT-DC allows us to (1) solve methodological problems resulting from studying categorical and continuous variables separately; (2) study discrete latent cognitive states (e.g., as in 2HT models) and the latent continuous variables associated with these states; and (3) estimate parameters of SDT models that include ordinal and continuous variables.

The main purpose of the present study is to highlight the advantages of incorporating quantitative variables in both 2HT and SDT models. More specifically, we will try to widen the range of research questions related to these models and to make more accurate conclusions about them. To achieve these goals, we use the data from Juola et al. (2019), in which continuous and categorical variables (RTs and confidence levels, respectively) were measured in a recognition experiment using words lists, and the proportions of targets were manipulated across trial blocks.

## Theoretical assumptions and modeling approach

The psychological assumptions about confidence ratings and RTs are different for the SDT and 2HT models. In the next section we will explain the theoretical assumptions of each model and, following the experimental design of Juola et al. (2019), we will describe their reparameterization as MPT-DC models.

## Classic two-high-threshold model

**Fig. 1:**
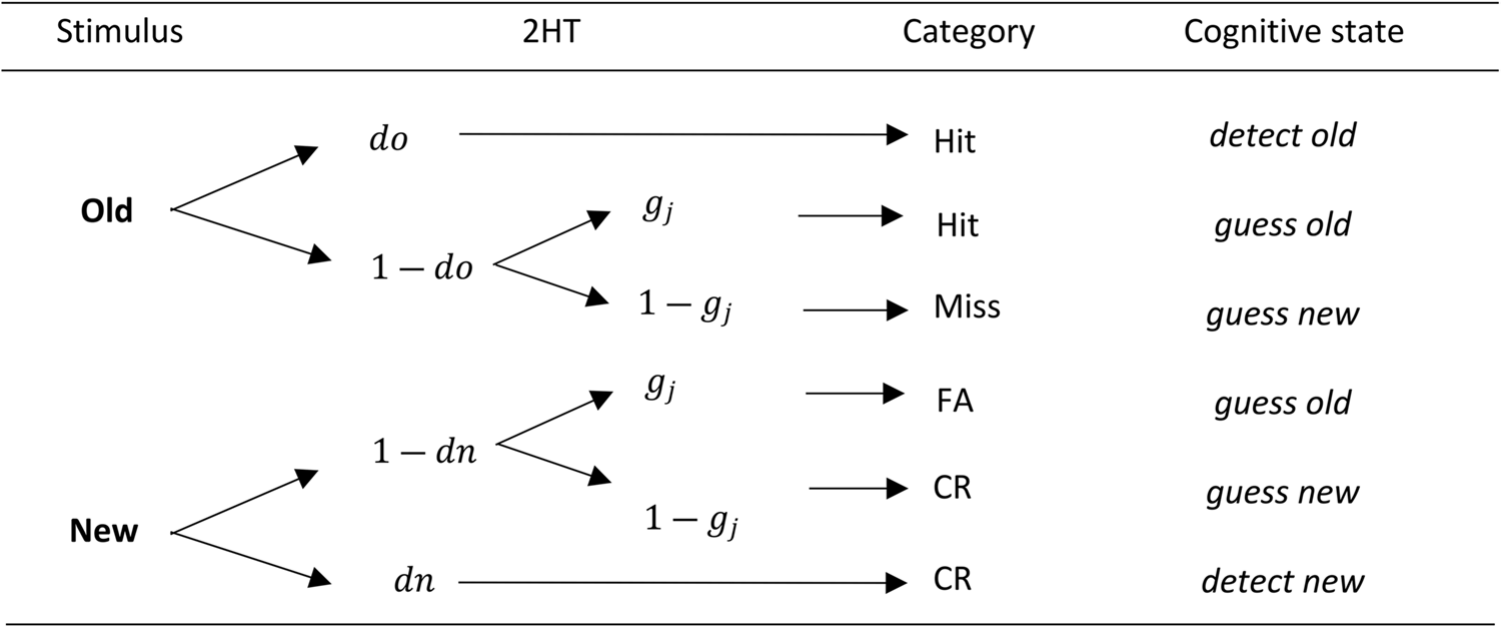
Classic 2HT model. *Note.* The left column refers to the presented stimulus whereas the 2HT model column includes the process probabilities. The responses produced by each 2HT branch are displayed in the category column (Hit/Miss/FA/CR), while distinguishable branches, which form cognitive states, are shown in the right column. Parameters *do*, *dn*, and *g* represent the probabilities of the *detect old*, *detect new*, and *guess old* processes, respectively. Subindex *j* of parameter *g*_*j*_ indicates the condition corresponding to relative target frequencies

Although the underlying variable of memory strength might be continuous, the 2HT model assumes that two thresholds exist that define three states, within each of which the cognitive experience is the same. One can experience relatively certain states of *detect new*, *detect old,* and an intermediate state of uncertainty that results in old or new guesses (see Fig. 1). These cognitive states are determined by a series of discrete processes whose probabilities can be estimated using MPT models (Bröder & Schütz, 2009).

As can be seen in Fig. 1, the *detect old* and the *detect new* states are determined by the probabilities of detecting old, *do*, and new words, *dn*, respectively. The high thresholds do not allow the *detect old* state to be entered on new word trials, nor are target trials allowed to result in the *detect new* state. The guessing states include both a nondetection probability and a guessing probability. Depending on whether old or new words are presented, the probability of nondetection will be the complementary of the detection probability for each tree (i.e., 1 − *do* for old words and 1 − *dn* for new words). The guess states result in a bias for saying “old” or “new” when the participant is not in a *detect old* or *detect new* state (Delay & Wixted, 2021; Malmberg, 2008). While the guess old state includes a guessing old probability, *g*, the guess new state includes a guessing new probability, 1 − *g*. Notice that in the current model the *guessing old* (*g*) and *guessing new* (1 − *g*) probabilities for old and new stimuli are the same. This assumption is based on previous studies (Juola et al., 2019), although with different theoretical assumptions, other restrictions could be established (Heck & Erdfelder, 2016; Heck et al., 2018b; Kellen & Klauer, 2014; Kellen et al., 2015). Also, because manipulating the proportion of targets has the assumed effect of changing the guessing bias, but not the detection probability, the probability *g* varies among the *J* experimental conditions defined by the proportions of targets, thus having *g*_*j*_ parameters. The expected effect is that *g*_*j*_ increases as the proportion of targets increases in any trial block.

## Confidence levels for the two-high-threshold model

CLs in 2HT models are given by a translation of memory states into confidence scales. However, the way this translation is done is not entirely straightforward. One possible assumption, called the certainty assumption, is that a detection state, for both old and new stimuli, can produce only high-confidence responses, whereas states of uncertainty (*guess old* or *guess new* states) can generate responses of all CLs. The certainty assumption has been widely criticized as poorly fitting the data and being unrealistic (e.g., Luce, 1963). In response to this issue, more flexible models have been generated, but these have been criticized for being too unconstrained and neither generalizable nor useful. Authors such as Bröder et al. (2013) suggest that model flexibility might be essential to grasp the actual complexity of some experimental paradigms, because the individual distribution of confidence ratings in a 2HT model may be influenced by many variables unrelated to the recognition system. Such nuisance variables, reflect the participants’ response styles (Henninger & Plieninger, 2021; Naemi et al., 2009) more than their detection abilities or guessing strategies.

In other words, the use of a 2HT model without too many constraints is motivated by the vast number of ways of responding and the various factors that influence the distribution of CLs. That said, here we will assume that any cognitive state can be the source of “high,” “medium,” and “low” confidence responses, but the distribution of CLs is conditioned by the cognitive states included in the 2HT model (e.g., confidence distributions in *detect* states are usually characterized by a higher probability of high- than of low-confidence levels).

**Fig. 2:**
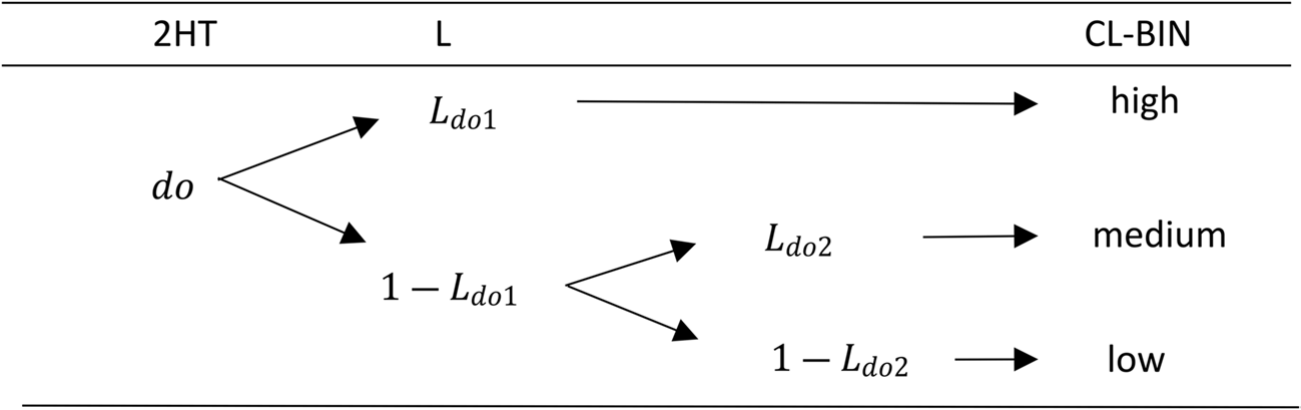
Confidence level and two-high-threshold model extension example. *Note:* The *do* parameter in the 2HT column represents the detect old state probability of the classic 2HT model. The *L* parameters characterize the probability of each confidence bin (CL-BIN) included in each state. The subscript “*do*” of the *L*_*do*_ parameter denotes that it is the *L* parameter associated with the *detect old* state. The probability for a high-confidence response will be, *L*_*do*1_, for medium-confidence, (1 − *L*_*do*1_) · *L*_*do*2_, and for low-confidence,(1 − *L*_*do*1_) · (1 − *L*_*do*2_)

To simplify the description of the 2HT model in which CLs are incorporated (2HT-CL), we exemplify the extension of the *detect old* state of the MPT-DC model, with probability *do* (see Fig. 2.). As in Juola et al. (2019) the CL is an ordinal variable with three levels, and the confidence bins (high, medium, and low confidence) have been included for each branch of the 2HT model. This implies that the total number of trials must be distributed among more subcategories. The new nodes will be associated with the *L* parameters,^1^ that model the probability of each confidence category and branch. Hence, for the *detect old* state the probability of a high-confidence response will be, *L*_*do*1_, for medium-confidence, (1 − *L*_*do*1_) · *L*_*do*2_, and for low-confidence,(1 − *L*_*do*1_) · (1 − *L*_*do*2_).

## Reaction time in the two-high-threshold model

Regarding RTs in 2HT models, it has been advocated that once the detection threshold is exceeded, faster responses occur compared with those found in nondetection states, which is compatible with the results of several studies (Heck & Erdfelder, 2016; Klauer & Kellen, 2018; Starn, 2021). To study the relationships between cognitive states and their RTs, a model that allows modeling latent RTs is needed.

Two procedures have been proposed to evaluate an MPT model in which a quantitative variable, such as RT, has been incorporated: parametric (Heck et al., 2018b; Klauer & Kellen, 2018) and nonparametric (Heck & Erdfelder, 2016). The parametric model is based on assumptions about the shape of the distribution of the quantitative variables and the parameters that characterize them (Heck et al., 2018b). Although it leverages and provides more information from the data than the nonparametric version, it may not be a suitable procedure for situations with important discrepancies between what is assumed and how the data are distributed (Klauer & Kellen, 2018). With respect to the nonparametric procedure, instead of analyzing the whole set of observed RTs, it assumes that any continuous variable can be discretized into bins and represented by a histogram (Van Zandt, 2000). Although it provides limited information about quantitative data, it has the advantage of requiring fewer assumptions about their distributions.

As far as usability is concerned, the nonparametric procedure may be easier to apply with a user-friendly library originally made for classical MPTs, called MPTinR (Singmann et al., 2022). This procedure enables fitting SDT models with continuous variables, something that, to the best of our knowledge, is still not allowed by libraries specifically built for fitting parametric MPT-DCs (Heck et al., 2018a; Klauer & Kellen, 2018).

**Fig. 3:**
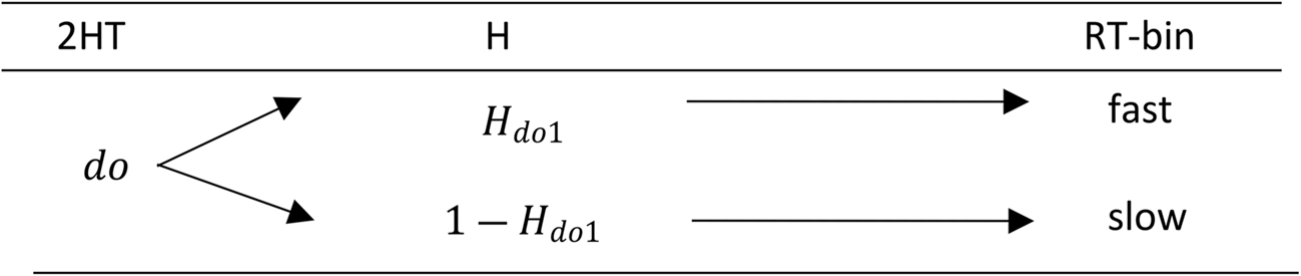
Reaction time two-high-threshold model extension example. *Note.* The *do* parameter on the 2HT column represents the *detect old* state probability of the classic 2HT model. The *H* parameters characterize the probability of each RT-bin included in each state. *H*_*do*1_ is the probability that a response triggered by the *detect old* state (subscript “*do*”) is fast, and a probability of 1 − *H*_*do*1_ that it is slow

Considering all the above, we will now explain the modeling procedure of RTs according to the nonparametric approach of Heck and Erdfelder (2016). To do this, the continuous variable is discretized into RT-bins and then the model is reparametrized, forming an extended-MPT model. In other words, the branches of the original MPT are subdivided into as many subbranches as RT-bins, which implies that the total number of observations must be distributed among the bins. Then a new probability parameter is assigned to each bin. In our work, the continuous variable RT has been divided into fast and slow bins. We exemplify the extension of the detect old state in Fig. 3.

## Confidence level and reaction time two-high-threshold model

**Fig. 4:**
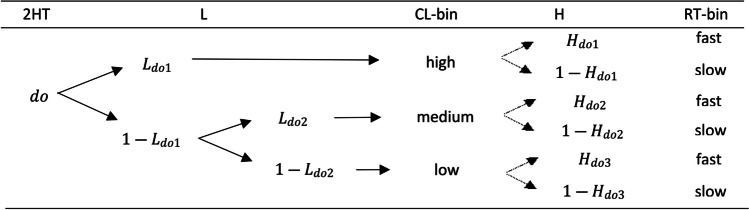
Confidence levels and reaction times for the two-high-threshold model extension example. *Note.* The 2HT column represents the *detect old* state probability of the classic 2HT model. The *L* parameters characterize the probabilities of entering each confidence bin (CL-bin) and the *H* parameters the probabilities of each reaction time bin (RT-bin) included in the *detect old* state

To study whether, as theory predicts (Juola et al., 2019; Murdock, 1985; Ratcliff & Murdock, 1976), higher CLs result in faster responses than lower CLs, we need to study the interactions between RTs and CLs, and thereby model these variables jointly. For this purpose, we can use the 2HT-CL model and, from each of the extended CL branches, add two new branches that are the result of discretizing the RT data into two bins, the *fast* and *slow* ones. These bins will have an associated probability *H* which is the probability of each RT bin given an extended CL branch. For example, by extending the old high-confidence detect state (see Fig. 4), there is the parameter *H*_*do*1_, which represents the probability that the high-confidence detect response is *fast*, and 1 − *H*_*do*1_ that it is *slow.*

## Classic signal theory detection model

**Fig. 5:**
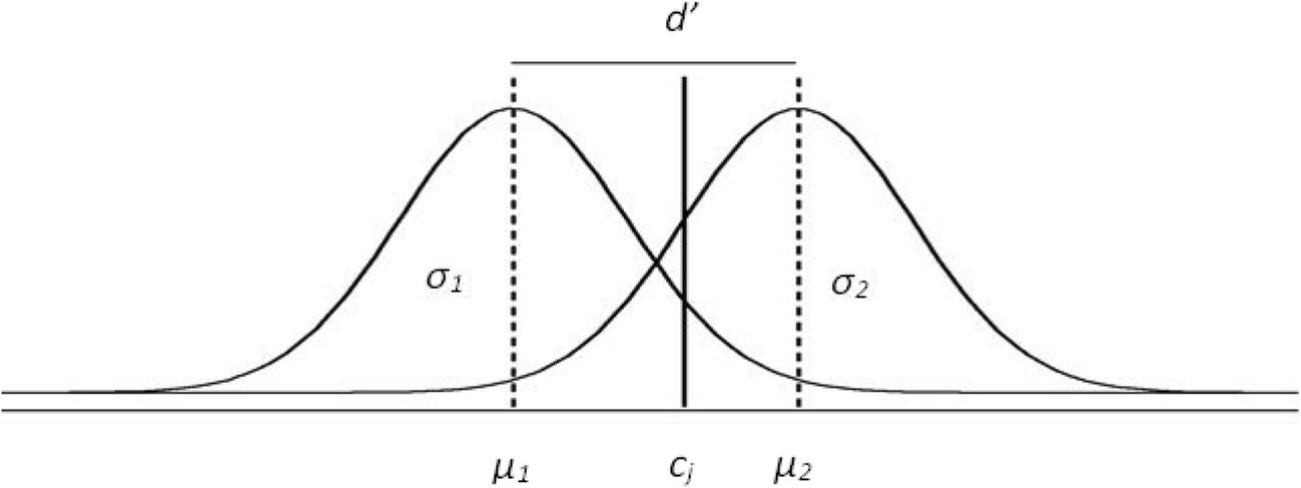
Signal detection theory model. *Note.* Signal detection model of recognition. The areas to the right of *c*_*j*_ in the target (right) distribution and the distractor (left) distribution represent Hit and FA probabilities, respectively. The areas to the left of *c*_*j*_ in the target and distractor distributions represent the respective probabilities of a Miss and a CR

We will now explain a continuous memory recognition model (i.e., the SDT model). According to this model, the test stimulus is translated by the observer into a continuous random variable of memory strength or familiarity. As can be seen in Fig. 5, the probability density functions of this random variable for old and new stimuli are assumed to follow two normal (i.e., Gaussian) distributions, respectively, that overlap to some extent (Macmillan & Creelman, 2004). Here the recognition model is described by the sensitivity parameter of the SDT, *d*^′^, and by the amount of variability in the familiarity (standard deviation), that could be *σ*_1_ and *σ*_2_, for new and old items, respectively, or just *σ* if homoscedasticity is assumed. The subject establishes a decision criterion, *c*, that allows him or her to divide the familiarity continuum into two regions, an “old” response region above *c*, and a “new” response region below *c*. Depending on experimental conditions *c* can vary. For example, it is expected that when the proportions of targets in a test block is increased, *c* decreases, while sensitivity should not change (Juola et al., 2019**)**. Then, as there are *J* = 3 experimental conditions differing in target proportions (65%, 50%, and 35%) there will be three parameters *c*_*j*_. Note that to reduce the number of parameters the mean of the new stimulus distribution, *μ*_1_, is set to 0, and its standard deviation, *σ*_1_, to 1, then: *d*^′^, the difference between the means of the two distributions is equal to *μ*_2_. Thus only *d*^′^, *σ* and *c*_*j*_ need to be estimated.

## Confidence in the signal detection theory model

**Fig. 6:**
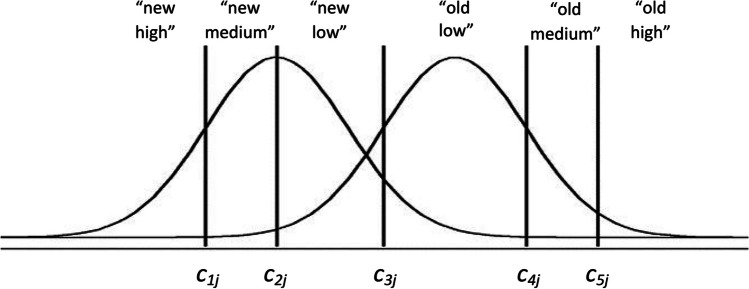
Signal detection theory confidence model. *Note.* Signal detection model of recognition with varying confidence levels. Each decision criterion *c*_*kj*_ along the familiarity continuum delimits the area or probability of CLs for new and old responses (from “new-high” to “old-high”) in both distractor (left) and target (right) distributions

The establishment of new criteria allows for capturing the distribution of CLs (see Fig. 6). The continuous process model assumes that confidence judgments are based on the establishment of a more specific set of decision boundaries that allows participants to give combinations of the type of stimulus and the level of confidence in their responses. The CLs given by each participant are simply graded “old”/“new” judgments made according to where the familiarity of each test stimulus is positioned (Bröder et al., 2013; e.g., in Fig. 6 a familiarity value between *c*_3*j*_ and *c*_4*j*_ would result in an “old-low” response). Thus, for modeling the Juola et al. (2019) experiment, where there are six possible answers (“old-high,” “old-medium,” “old-low,” “new-low,” “new-medium,” and “new-high”) since *K* = 5, criteria *c*_*kj*_ are included. Note that the *j* subscript of *c*_*kj*_ implies that the manipulation of the target proportions (*j*) affects all criteria. Nevertheless, if, as expected, the response criteria change monotonically with the relative target frequency blocks (Juola et al., 2019), then, the *j* conditions affect the *c*_*k*_ parameter by the same amount. That is, although the manipulation of target proportion alters the position of all response criteria, the distance between different *c*_*k*_ is assumed to be the same under the *j* conditions. Therefore, only the *K* − 1 neighborhood distances between criteria (∆*c* = *c*_*k*_ − *c*_*k* − 1_) will have to be estimated, such that,

1 $${c}_{2j}={c}_{1j}+\Delta {c}_2,$$

2 $${c}_{3j}={c}_{1j}+\Delta {c}_2+\Delta {c}_{3,}$$

3 $${c}_{4j}={c}_{1j}+\Delta {c}_2+\Delta {c}_3+\Delta {c}_{4,}$$

4 $${c}_{5j}={c}_{1j}+\Delta {c}_2+\Delta {c}_3+\Delta {c}_4+\Delta {c}_5.$$

## Reaction time in the signal detection theory model

Due to the direct relationship with SDT models, confidence has been extensively studied; however, RTs tend to be ignored. The strength theory is precisely an attempt to theoretically relate SDT models and RTs. It suggests that the closer the evidence of familiarity of the stimulus is to the criterion, the longer the RT (Emmerich et al., 1972; Malmberg, 2008; Murdock, 1985; Norman & Wickelgren, 1969). To test these hypotheses, we propose to employ MPT-DC models.

**Fig. 7:**
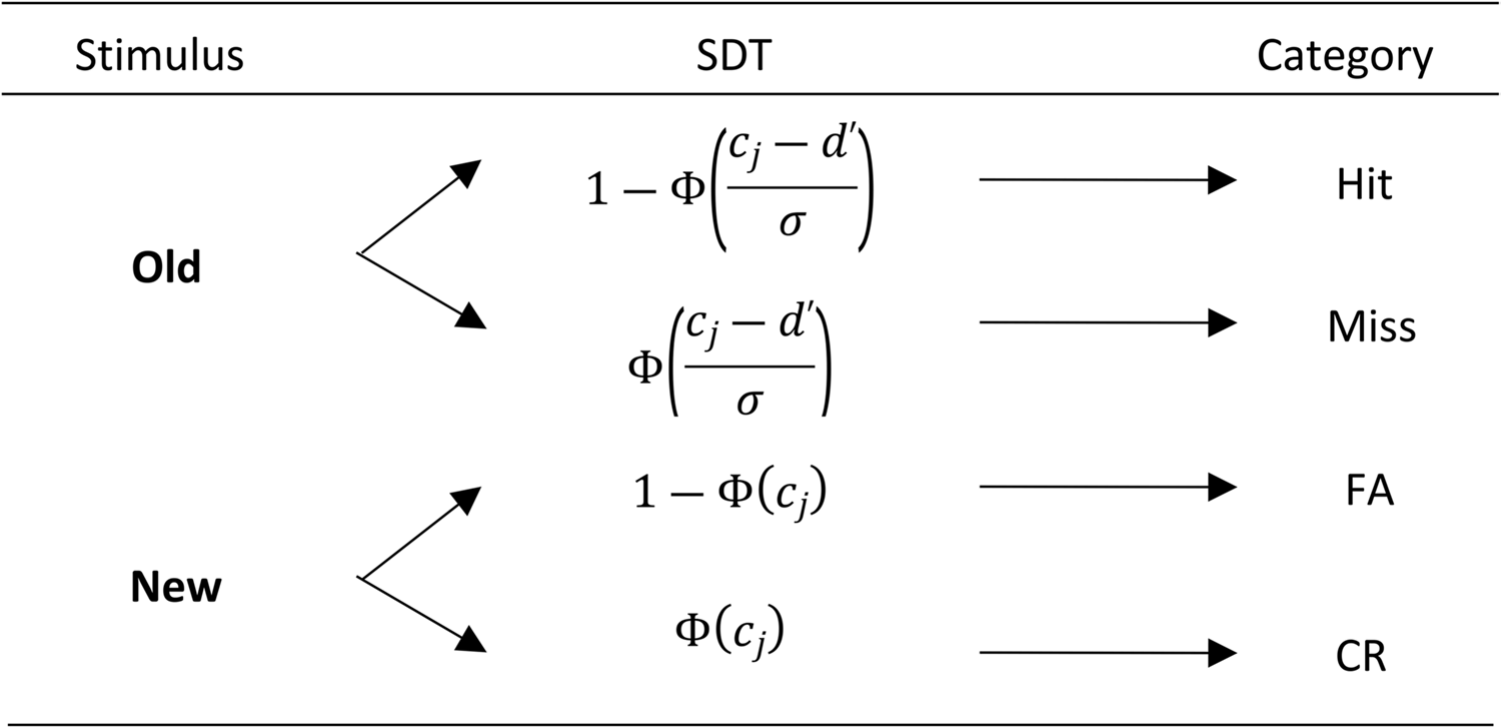
Signal detection theory model. *Note.* The left column refers to the presented stimulus whereas the SDT model column includes the probability for each category. The responses produced by each SDT model branch are displayed in the category column (Hit, Miss, FA, CR)

For building an SDT model in which RTs are incorporated, the SDT model is first reparametrized as a multinomial model (see Singmann & Kellen, 2013). Figure 7 shows the reparametrized SDT model, in which the branches of the multinomial model represent different response categories and the probability of each category is a function with typical SDT parameters. The parameters included in the table are *d*^′^, which represents the sensitivity, and *σ* the variability of the signal distribution ( *μ*_1_ is set to 0 and its standard deviation, *σ*_1_, to 1; see Footnote 3). The cumulative normal distribution is represented by Φ.

**Fig. 8:**
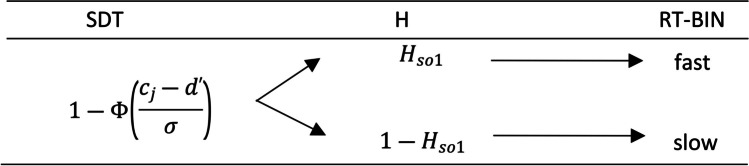
Reaction time signal detection theory model extension example. *Note.* The SDT column represents the Hit category probability of the classic 2HT model. The *H* parameters characterize the probability of each reaction time bin (RT-BIN) included in the example

Once the SDT is reparametrized, we can follow the previously used logic for the inclusion of the *H* parameters associated to the probability of each RT-bin (see Fig. 8 for an example of RT inclusion in the SDT model for the first branch). Again, the *H*_*so*1_ is the probability for *fast* responses and 1 − *H*_*so*1_ for *slow* ones. The subscript “so” has been added to the *H* parameter in Fig. 8 to refer to the first branch of the SDT model where a signal is presented, and the response is “old.”

## Confidence and reaction time in the signal detection model

As noted above, strength theory (Emmerich et al., 1972; Murdock, 1985; Norman & Wickelgren, 1969) suggests that more extreme familiarity (high-confidence responses) results in shorter RTs. Consequently, there is an inverse relationship between confidence and RTs. Indeed, results from numerous studies have shown an inverted U-shaped relationship between familiarity and latencies, and a U-shaped relationship between familiarity and confidence judgments, which is compatible with strength theory (Baranski & Petrusic, 1994, 1998; Murdock & Dufty, 1972; Ratcliff & Starn, 2009; Starn, 2021; Weidemann & Kahana, 2016). However, it does not capture whether the relationship differs between correct (CR, Hit) and incorrect (Miss, FA) responses, and would imply an inverse relationship between RTs and CLs for all categories, which may not be a feasible prediction.

In short, to disentangle this issue, it is necessary to build models that allow the relationships among response categories, CLs, and RTs to appear.

**Fig. 9:**
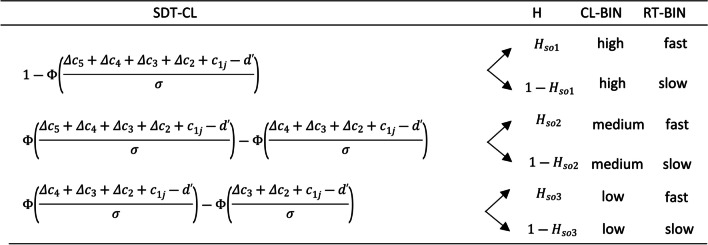
Confidence levels and reaction times in the signal detection theory extension model example. *Note.* The SDT-CL column represents the hit category probability of the SDT model, for high (top row) medium (middle row) and low (bottom row) CLs. The *H* parameters characterize que probabilities of each reaction time bin (RT-BIN) included for the hit category

In order to develop a more general model of CLs and RTs, we propose to use a reparametrized SDT confidence model, SDT-CL, and then include RTs by means of an MPT-DC. The parameters included in the SDT-CL are those already referred to for a transformed SDT model parameterized as a multinomial model (see Fig. 7) and, additionally, *Δc*_1_, *Δc*_2_, *Δc*_3_, *Δc*_4_and *Δc*_5_, the *K − 1* increments of criterion (∆*c*) (see Equations 1–4). Having done the above, as shown in the example in Fig. 9, it would suffice to include the *H* parameters that characterize the RT-bins of the SDT-CL model.

In summary, with the described MPT-DC procedure we can (1) fit classic 2HT and SDT models of recognition memory (without CLs and/or RTs), (2) either include the CLs (by aggregating the *∆c* parameters or *L* parameter, for SDT and 2HT classical models, respectively), (3) or RTs (by aggregating the *H* parameters to the classical models), (4) or include all variables simultaneously. We will work with all these versions of such models with the aim of studying the methodological and theoretical advantages of including none, one, or both aforementioned variables.

## Method

### Data availability

The data have been collected from https://osf.io/y78mk/. Detailed information on the participants, materials and experimental procedure can be found in Juola et al. (2019). The following is a summary of these sections:

- PARTICIPANTS: The data were obtained from 47 students and members of the academic community from the Universidad Autónoma de Madrid.
- MATERIALS: The study used 500 common Spanish nouns. These words were displayed one at a time on a computer monitor during the study and test phases. The experiment was conducted using E-Prime 3, a software tool specifically designed for psychological research.
- EXPERIMENTAL PROCEDURE: The participants were presented with a study list consisting of 250 words, displayed one at a time, and they were instructed to pronounce each word as it appeared. Following the completion of the study phase, participants engaged in a short intervention task followed by five blocks of test trials. Each block consisted of 100 words, and the target proportions in each block were varied between .15 and .85. Participants were informed in advance of these proportions and were told to respond as rapidly as possible with an old/new response while being careful to avoid errors. After the response, they were asked to indicate their confidence on a 3-point scale indicating whether they were certain, relatively certain, or uncertain of the accuracy of their response. The entire session lasted less than 1 hour. In analyzing the data, we became aware that the most extreme manipulation conditions, when the proportions of targets were .15 or .85, seriously affected the goodness of fit of the models. This was likely due to these conditions yielding parameter estimates that were close to the boundaries of the parameter space (e.g., guessing probabilities very close to either 0 or 1; Silvapulle & Sen, 2005). Therefore, we decided to restrict our analysis to the blocks with target proportions of .35, .50, and .65.

### Model details

Four separate analyses were conducted to study: (1) classic SDT and 2HT recognition models with only the accuracy measures; (2) 2HT and SDT models with CL bins (2HT-CL and SDT-CL, respectively); (3) 2HT and SDT with RT bins (2HT*-*RT and SDT-RT); and (4) 2HT and SDT with both CL and RT bins (2HT-CL-RT and SDT-CL-RT, respectively).

Models were built using a nonparametric procedure (Heck & Erdfelder, 2016) with the discretization of the continuous RTs into bins set individually for each subject using the geometric mean of their RTs. If the RTs exceed this criterion, they were assigned to the slow bin, while those below the criterion were assigned to the fast bin. The criterion was set using data-dependent RT boundaries, namely the log-normal approximation, which is the default recommendation extrapolated from previous simulation studies. The procedure consists of log-transforming the RTs, calculating the mean and variance, obtaining the quantiles, and then converting them back to the standard RT scale to obtain the necessary cut-offs for categorization (see log-normal approximation procedure and simulations in Heck & Erdfelder, 2016).

Both 2HT and SDT classic versions include five parameters. The 2HT model includes two detection parameters, *do* and *dn,* and *J* = 3 guessing parameters, *g*_*j*_, one for each target proportion manipulation condition *j*. The SDT model includes one sensitivity parameter, *d*^′^, one variability parameter, *σ,* and *J* = 3 criteria, *c*_*j*_.

The 2HT-CL model contains 13 parameters, which are five from the classical version, plus the *L* parameters that define the three CL-bins for *detect old*, *detect new*, *guess old*, and *guess new* states. The SDT-CL model includes nine parameters, the five previously indicated in the SDT model plus the *K* − 1 = 4 criterion increments (∆*c*) that allow estimating the distances between the different criteria for response confidence ratings.

The 2HT-RT model contains nine parameters, the five classic parameters plus the *H* parameter that defines the two RT bins of the *detect old*, *detect new*, *guess old*, and *guess new* states. The SDT-RT model contains nine parameters, the five classic parameters plus the *H* parameter that defines the two RT-bins for Hits, Misses, FAs, and CRs.

In the 2HT-CL-RT model 25 parameters were included. The 13 indicated in 2HR-CL plus the *H* parameters that define the probabilities for the two RT bins per each extended branch of the 2HT-CL model. The SDT-CL-RT model has 21 parameters, the nine from SDT-CL plus the *H* parameters that define the probabilities of the two RT bins per branch.

### Fits

All models were fitted using MPTinR (Singmann et al., 2022) for R Core Team (2022) and their formulation is available in Appendix A Tables 6, 7, 8, 9, 10, 11, 12 and 13. The *SE* (standard errors) of the estimated parameters is obtained directly from MPTinR using the Hessian matrix. For assessing goodness of fit, we initially employed the Pearson chi-squared (χ^2^) goodness of fit test. However, due to the presence of low expected frequencies in some cells of the fitted models, the use of chi-squared tests is not recommended, since the asymptotic properties are not preserved (Langeheine et al., 1996; Lin et al., 2015). As an alternative, we adopted a parametric bootstrap goodness-of-fit test. To do so, we generated 1,000 parametric bootstrap samples for each to-be-tested model and subject, fitting the corresponding model to each of these samples and calculating χ^2^. From each bootstrap-generated χ^2^ distribution, we obtained the critical value. To evaluate the statistical significance, we compared the estimated chi-squared value derived from fitting the observed data with the critical value. If the estimated χ^2^ value was less than or equal to the critical value, we retained the null hypothesis, indicating that the differences between observed and expected frequencies were not statistically significant, thus concluding that the model provided a suitable fit to the data.

In addition, since the ratio between the data (*n*) and the number of parameters (*p*) is relatively low, a corrected *AIC* has been calculated (Burnham & Anderson, 2002) which follows the expression below:

$$AI{C}_C= AIC+\left[{~}^{\left(2\cdot p\cdot \left(p+1\right)\right)}\!\left/ \!{~}_{\left(n\hbox{--} p\hbox{--} 1\right)}\right.\right].$$

### Analysis

To determine whether parameter estimations could be improved by adding more dependent variables, we found the standard errors (*SE*) of the estimated parameters of the SDT and 2HT models without and then with the inclusion of CL (2HT-CL and SDT-CL), RT (2HT-RT and SDT-RT) and with the simultaneous inclusion of both CL and RT data (2HT-CL-RT and SDT-CL-RT). The results of this analysis can be found in the first results section.

In the second results section we assess the scope of research questions that can be covered using MPT-DC models, fitting 2HT and SDT models that jointly model CLs and RTs. Descriptive analyses were performed on raw aggregated data (see Appendix B Tables 14, 15 and Fig. 12), while statistical comparisons for each model were made on individual estimated parameters. As for the analyses of the parameter predictions, we will only mention those related to the interactions between latent branches (cognitive states or categories), CLs and RTs. Details concerning all analyses performed and their predictions can be found in Appendix C Figs. 13, 14, 15, 16 and 17.

In the third results section we address the continuous-process versus discrete-process questions to demonstrate that the CL and RT evidence might alter conclusions about models’ performance. Individual comparisons between SDT and 2HT models were made using the Akaike information criterion (AIC) obtained by fitting the models to experimental data from Juola et al. (2019). Cross tabulations were constructed to analyze changes in the selection of the best individual model (in the comparison between the SDT vs. 2HT models) based on the variables included in these models. The *χ*^2^ test of independences was applied to each crosstab, where Phi (*φ*) was used as a measure of effect size. Significance level alpha was set to .05.

## Results

### Accuracy analysis

**Fig. 10:**
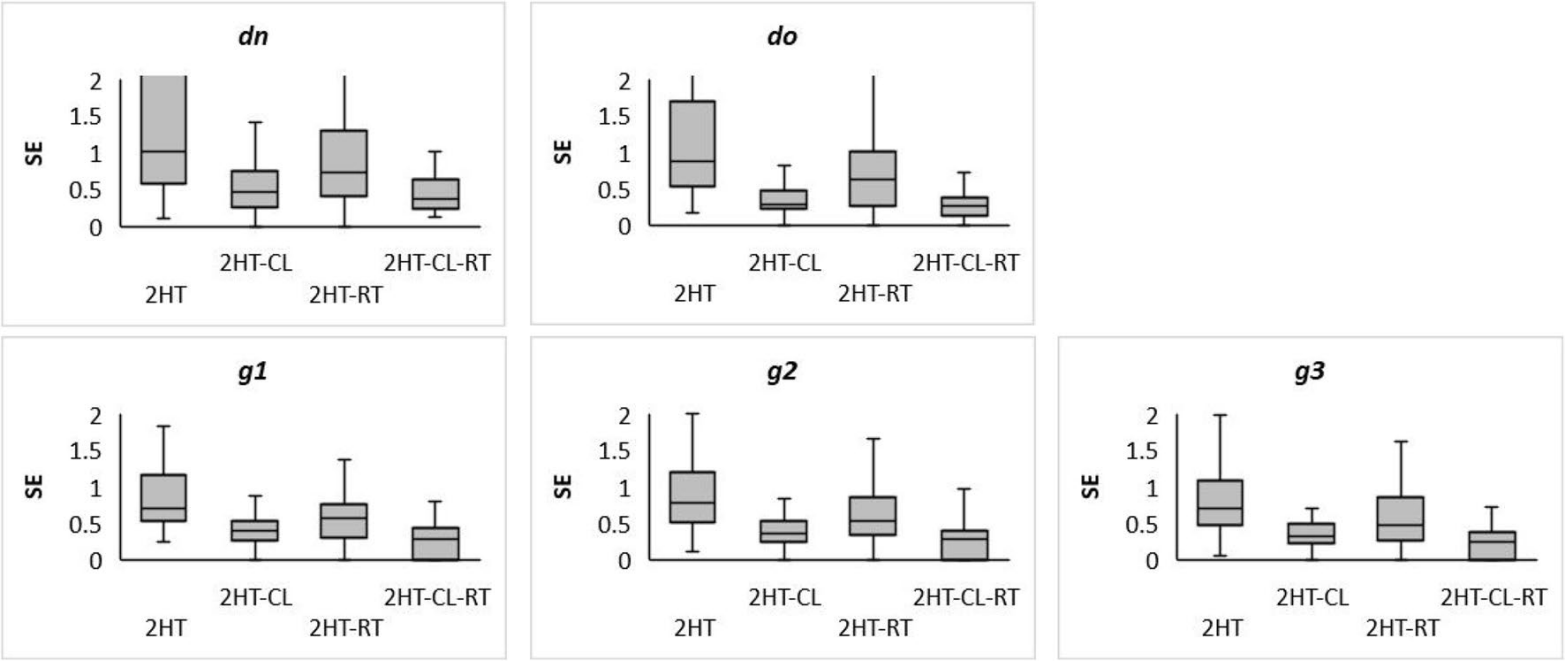
2HT individual standard errors for detect new, detect old, and guess old probability parameter *estimates. Note.* Box and whisker plots for the SEs of the estimated parameters in the 2HT models. The bottom and top sides of the boxes are the first and third quartiles while the horizontal lines inside the boxes are the median *SE* values. The lower and upper limits of the whiskers represent ±3 standard deviations, respectively

**Fig. 11:**
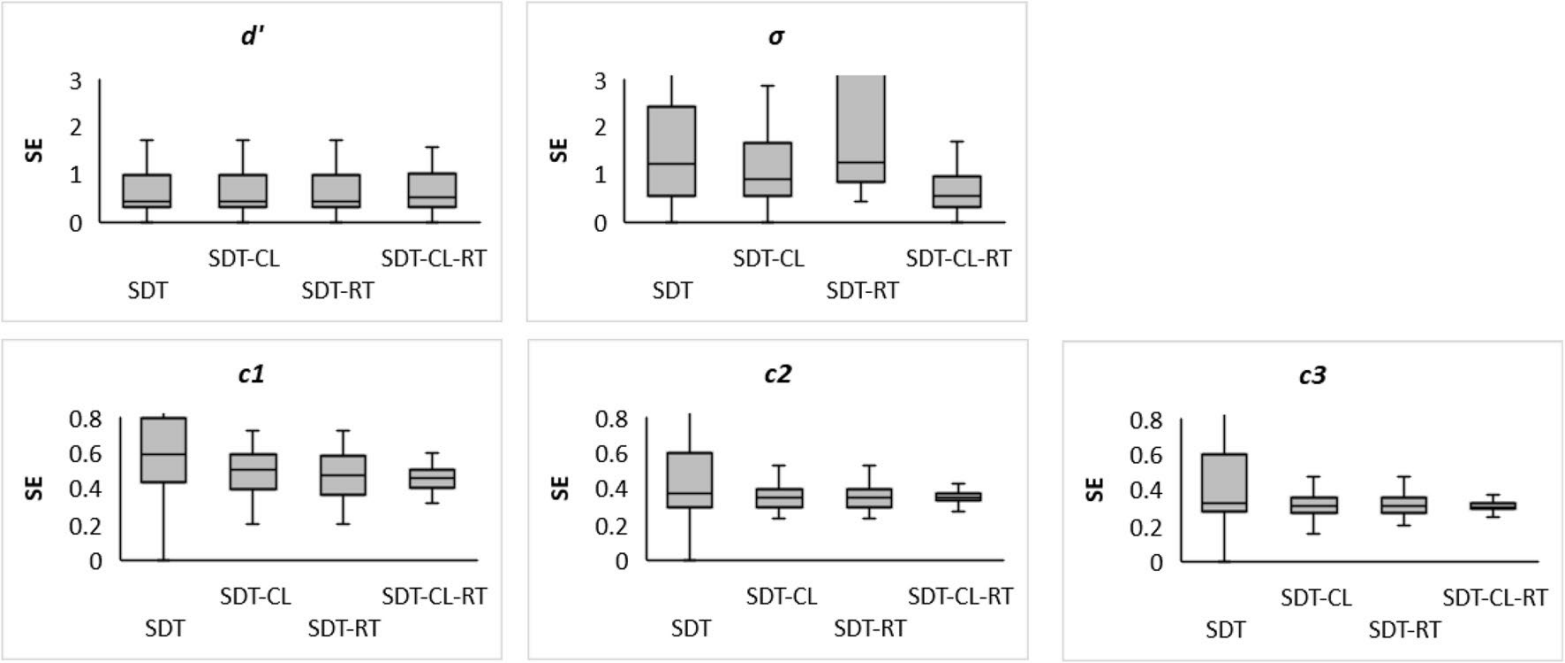
SDT individual standard errors for the sensitivity, variability, and decision criteria parameters. *Note.* Box and whisker plots for the *SE*s of the estimated parameters in the SDT models. The bottom and top sides of the boxes are the first and third quartiles while horizontal lines inside the boxes are the median *SE* values. The lower and upper limits of the whiskers represent ±3 standard deviations, respectively

**Fig. 12:**
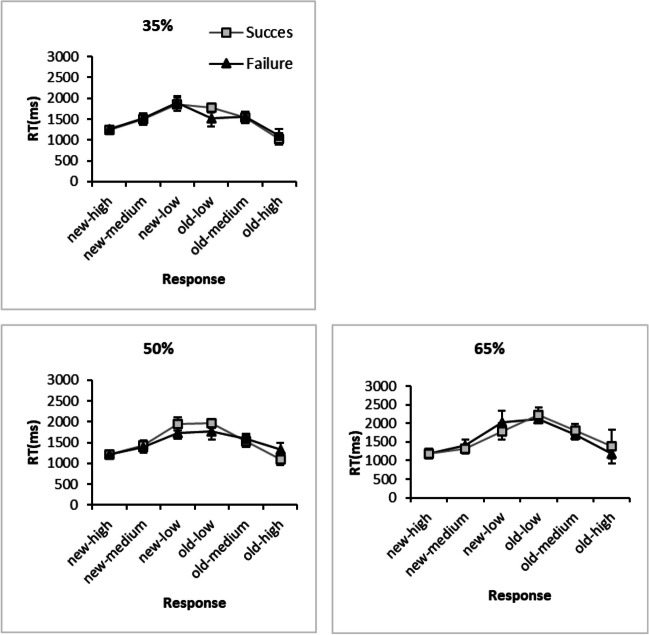
(Inverse U-shape) relation between RT and confidence ratings. *Note*. Mean RT (ms) for all subjects, *N* = 47, is shown for each response-confidence combination (new-high/new-medium/new-old/new-low/old-low/old/medium/old-high), and response-confidence responses are ordered by familiarity, from extreme low familiarity (left) to extreme high familiarity (right). The different-colored lines allow distinguishing whether the result of the answer was correct or incorrect. Successes are in blue and failures in red. Each figure represents a different target proportion condition (35% to 65%). The error bars represent the standard errors of the mean (*SEM*) RTs.

Figure 10 shows box and whisker plots for individual *SE*s for the estimated parameters of the 2HT model. Despite a slight decrease in *SE*s for the $\hat{dn}$, $\hat{do}$, and $\hat{g_j}$ parameters with the inclusion of CL parameters, we observed a clear decrease of *SE*s when CL and RT were simultaneously analyzed. Regarding the SDT model (see Fig. 11), we observed a slight reduction of $\hat{\sigma}$ and the $\hat{c_k}$ *SE*s when including both CL and RT.

In summary, by jointly modeling accuracy, confidence and speed, the standard errors of the individual parameter estimates have been reduced, especially in 2HT, but also in SDT.

### Discrete state versus continuous process

Table 2 displays the percentage of subjects for whom the null hypothesis of the *χ*^2^ bootstrapped test was rejected (see Appendix D Tables 16, 17, 18 and 19 for individual *χ*^2^ estimations and critical values obtained through bootstrapping). Based on bootstrapped chi-squared tests, we conclude that there is apparently no difference in the percentage of subjects with acceptable fits between the SDT and 2HT models. In particular, of the 47 subjects, only two (4.3%) showed a lack of fit to the classical models. This percentage increases when additional DVs are added, reaching almost 60% inadequate fits when both RTs and CLs are included. In principle, this could lead us to think that the choice of the classical versions of these models would be the most appropriate. As we will see in the results derived from the simulations, goodness-of-fit tests are not a good indicator of the relevance of the DVs when what really interests the researcher is the comparison between models.

**Table 2:** Frequency of hypothesis rejection of the bootstrap goodness-of-fit test

Model	2HT	SDT
Accuracy	2 (4.26%)	2 (4.26%)
CLs	25 (53.2%)	21 (44.7%)
RTs	24 (51.1%)	25 (53.2%)
CLs+RTs	27 (57.4%)	28 (59.6%)

*Note.* The first column indicates the 2HT and SDT model versions, the second and third columns indicate the frequency and percentage of subjects in which the null hypothesis of the *χ*^2^ bootstrapped goodness-of-fit test is not rejected, for the 2HT and 2HT model, respectively.

Regarding whether recognition memory is best explained by a continuous process or by discrete states, we find that, for Juola et al.’s (2019) experimental data (see Table 3), when not considering CLs nor RTs, there are more individuals that are better fit by the continuous SDT model (72.3%) than for the discrete 2HT model (27.7%). The same is true when considering RTs alone, but here the discrepancy of selection frequency in favor of SDT models is slightly smaller (70.2%). The same holds true when CLs are considered. In these situations, there is even a higher selection of the SDT model as the better one (80.9% for CL and 87.2% for CL+RT). The *AIC*_*C*_ values for each subject and model can be found in Appendix D Tables 16, 17, 18 and 19.

**Table 3:** Best model selection

**Model**		**n.par**	**AIC.best**	**%AIC.best**
Classic	SDT	5	34	72.3
	2HT	5	13	27.7
CL	SDT	9	38	80.9
	2HT	13	9	19.1
RT	SDT	9	33	70.2
	2HT	9	14	29.8
CL+RT	SDT	21	41	87.2
	2HT	25	6	12.8

*Note.* SDT vs. 2HT model comparisons are made by using classic model versions (Classic rows), that also include confidence levels data (CL rows), reaction time data (RT rows), and that uses all mentioned data (CL+RT rows). The number of parameters included in each model appears in the n.par column. The number of times and the percentage of best fit selection appears in the AIC.best columns.

Considering the cross tables of model selection shown in Table 4, we observe that model selection when accounting for CLs or both CLs and RTs (CL+RT) tends to coincide. Specifically, we can observe in Table 4 (A) that in matching selections made between models with CL and CL+RT, 38 subjects’ data are best classified by an SDT model and six by a 2HT model, with only three subjects changing their best model classification. In fact, we reject the null hypothesis of independence between selections made for models including CLs and those including CL+RT, with a considerably larger effect size compared with the other tests of independence (see Table 5 for tests of independence). We also observe in Table 4 (E) and (F) that the hypothesis of independence between selections made with RTs and those made with CLs or CL+RT, respectively, is rejected, although the size effect is not as strong as in Table 4 (A). In other words, model selections made with CLs or CL+RTs are related to those made when including RTs, but there are still quite a few inconsistencies between these classifications. Specifically, the number of subjects with incongruent classifications increases to 11 when comparing the selections made with models that include RTs and those that include CLs+RTs, and to 12 when considering models that include RTs and models that include CLs. Finally, when we analyze the classifications made with classical versions of the models, Table 4 (B), (C), and (D), we maintain the null hypothesis that selections made between classical models and any other version are not related to each other.

**Table 4:** Crosstabs for selection of the best model according to the variables included

**A**		CL+RT		Total	**B**		RT		Total
		SDT	2HT				SDT	2HT	
CL	SDT	38	0	38	Classic	SDT	26	9	35
	2HT	3	6	9		2HT	7	5	12
Total		41	6	47	Total		33	14	47
**C**		CL+RT		Total	**D**		CL		Total
		SDT	2HT				SDT	2HT	
Classic	SDT	29	6	35	Classic	SDT	27	11	38
	2HT	12	0	12		2HT	8	1	8
Total		41	6	47	Total		35	12	47
**E**		CL+RT		Total	**F**		CL		Total
		SDT	2HT				SDT	2HT	
RT	SDT	31	2	33	RT	SDT	30	3	33
	2HT	10	4	12		2HT	8	14	14
Total		41	6	47	Total		38	9	47

*Note.* Crosstabs for model selection are made between the following model version comparisons: CL and CL+RT model (A), Classic and RT (B), Classic and CL+RT (C), Classic and CL (D), RT and CL+RT (E), and RT and CL (F). The cells show the number of times a model was selected as the best one.

**Table 5:** Test of independence for selection crosstabs

*Crosstab*	*χ*^2^	*gl*	*p*	*φ*
A	29.41	1	>.001	.786
B	1.09	1	.287	.297
C	2.36	1	.125	.224
D	1.22	1	.27	.161
E	4.47	1	.034	.308
F	7.085	1	.013	.393

*Note.* The values of the *χ*^2^ statistic of the tests of independence between model selection patterns found in Table 5 are in the second column, while each row of the first column indicates the crosstabs on which the test is performed (from A to F). The following columns present the degrees of freedom (*gl*), the *p* value (*p*), and the effect size (*φ*) of the test.

## Discussion

### Accuracy improvements

In this section we will discuss the benefits of jointly analyzing different dependent variables in 2HT and SDT models. When we only use the frequency of each category of responses (Hit/Miss/FA/CR) we may lose information regarding other variables intrinsically linked to our experimental design.

Our results indicate that, indeed, the modeling of category frequencies with their confidence ratings and RTs together reduces *SE*s and thus improve the precision of the classical parameter estimates of the 2HT and SDT models. However, the informativeness is fully dependent on the variables to be included in each model. In our case, including RTs alone does not improve the precision of the estimates. This does not mean that the RTs do not reduce *SE*s, but only that they do so in conjunction with CLs. On the other hand, including CLs leads to a slight reduction of the *SE*s, which means that the precision of the joint estimation of CLs and RTs is greater than that of the separate estimations of the two variables.

## Recognition memory predictions

The SDT and 2HT models in their classical versions do not allow the simultaneous study of RTs, CLs, and accuracy.

To work with these variables authors such as Juola et al. (2019) performed three separate analyses to study the effects of (1) experimental manipulation of target rates; (2) clustering of responses into confidence bins; and (3) clustering of responses into RT bins. This has a certain limitation. First, CLs and RTs are not studied as dependent variables, so we cannot study the interaction between dependent variables, nor can we estimate latent CLs and RTs (e.g., separate estimates for RTs of *detect old* and *guess old* states). Second, trials from different blocks of relative target frequency are clustered in the same CL and RT bins and may interact in a masked way with the estimates from each model. Third, doing multiple separate analyses increases the risk of Type I errors and may encourage reporting only partial information when one variable has significant effects, but the other does not. The use of MPT-DCs models, however, allows us to overcome the above methodological difficulties while studying the compatibility of the theoretical hypotheses reported in the literature.

**Fig. 13:**
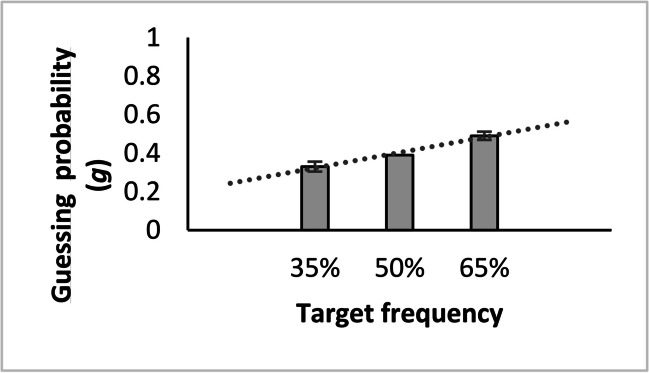
Effect of target proportion manipulation on guessing old probability. *Note.* The guessing old process probability is represented through three relative target frequency blocks. The error bars correspond to the 95% confidence interval (CI) of the *g* parameter estimate

**Fig. 14:**
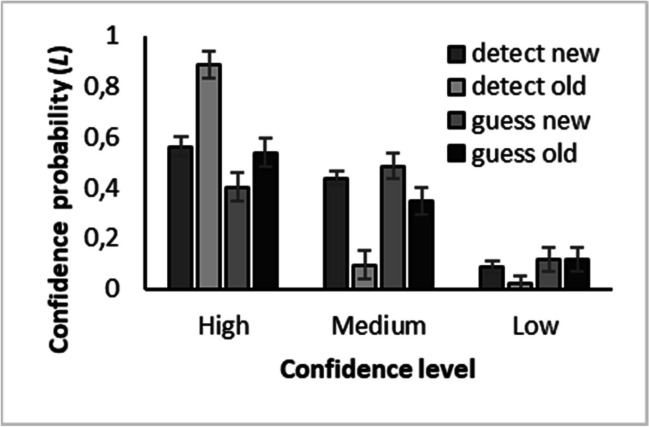
2HT state × confidence-levels effect. *Note.* The probability for high (left), medium (center) and low (right) CLs is shown for each of the 2HT discrete states (left to right bars: detect new, detect old, guess new, and guess old states). The error bars correspond to the 95% CI of the *L* parameter estimate

**Fig. 15:**
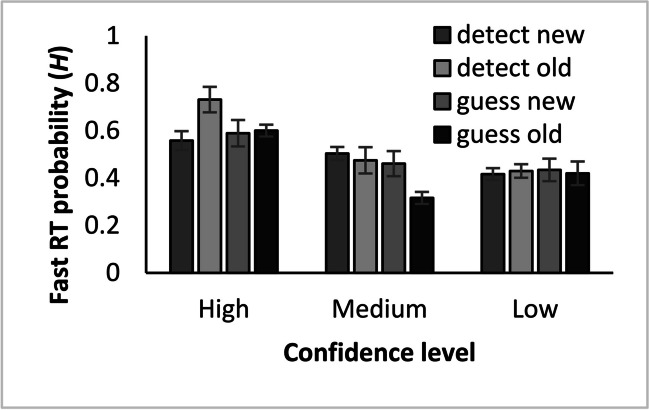
2HT confidence-level × short RT × state effect. *Note.* The probability for short RTs is shown for high (left), medium (center) and low (right) confidence ratings of each 2HT discrete state (left to right bars: detect new, detect old, guess new, and guess old states). The error bars correspond to the 95% CI of the *H* estimate

**Fig. 16:**
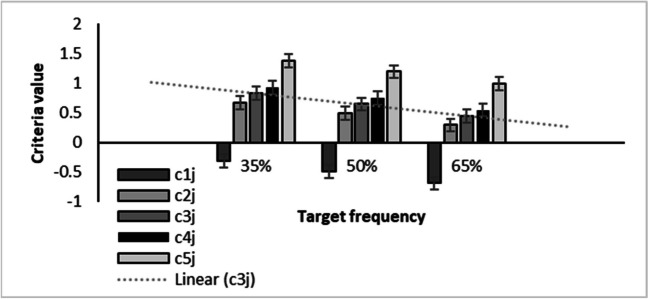
Effect of target proportion manipulation on decision and confidence criteria. *Note.* The *c*_*kj*_ parameters (c1j–c5j) are shown for each *j* relative target frequency blocks (35%–65%). A linear regression between target proportion and the decision criteria is represented by a dotted line. The error bars correspond to the 95% CI of the *c*_*kj*_ estimate

**Fig. 17:**
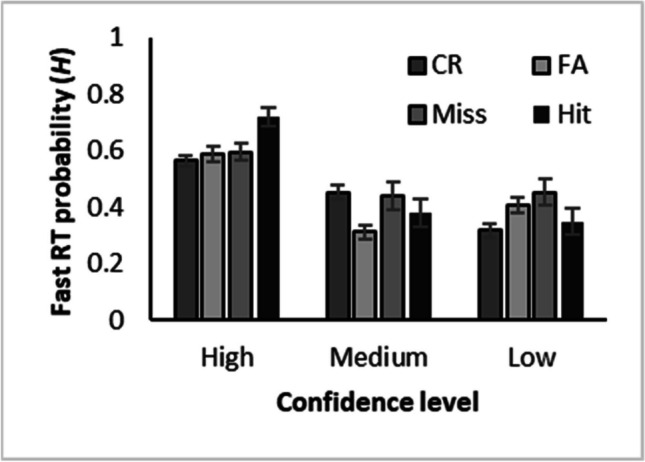
SDT confidence x fast RT x state effect. *Note.* The probability for short RTs is shown for high (left), medium (center) and low (right) CLs for each response category (left to right bars: detect new, detect old, guess new, and guess old states). The error bars correspond to the 95% CI of the *H* estimate

As expected, we found that the probability of guessing old, for the 2HT model, and the decision criterion between “new”/“old” responses, for the SDT model, changed monotonically with relative target frequency; the former being a direct relationship and the latter an inverse one. This pattern agrees with the analyses of the effect of aggregating data according to the relative frequency of targets in the Juola et al. (2019) study, in which the value of *g* decreased and the criterion values ( *c*_1_ to *c*_3_) shifted from left to the right as the target frequencies decreased (see Appendix C Figs. 13, 14, 15, 16 and 17).

The 2HT-CL-RT model was consistent with evidence relating detection responses to high confidence. High-confidence responses were faster than medium or low-confidence responses. However, when we separated this confidence probability distribution by states, we found some peculiarities. For example, the inverse relationship between RTs and CLs holds for all states but *guess old* states, where we actually find shorter RTs in low-confidence responses than in medium confidence responses. Note that these results could not be detected without a model capable of studying the interactions between the RTs of CLs and latent cognitive states (*detect old*/*detect new*/*guess old*/*guess new*).

As for the SDT model, some of the expected interaction trends were also not fulfilled. For instance, there is a clear pattern of faster responses at high CLs than at medium and low CLs in all categories except FA. That is, we again observe a different pattern of responses between “new”/“old” decisions.

Thus, the distribution of response times depends on the confidence level and the cognitive state (in 2HT) or response type (in SDT), and this result contradicts the idea that RT and CLs are measuring the same thing (Thomas & Myers, 1972; Weidemann & Kahana, 2016). These results also demonstrate the need for us to estimate the variables jointly to unravel the complexity of the relationships between them.

### Discrete-state versus continuous-process dilemma

The debate on whether recognition processes are discrete or continuous has been carried out with the classical versions of the SDT and 2HT models. Now that we have the data with MPT-DC models, with increased validity and the integration of continuous and discrete variables, we may draw a different and more complete set of conclusions.

The result of comparing discrete and continuous models is different if we treat quantitative variables as dependent variables, as proposed here, or as factors, as in Juola et al. (2019). What we do find in common is that it does not appear that all subjects have the same model of best fit, and that the proportions of subjects who follow a continuous or discrete model vary when different variables are considered.

These variations can have implications for comparisons between non-nested models, such as SDT and 2HT. Let’s consider a scenario in which an experimenter compares the SDT and 2HT models when RTs alone are included. In this case, if only the AICs of both models are compared, it could be concluded that there is an important number of subjects that fit the 2HT model better than the SDT model (27.7%). However, as discussed later, this last conclusion would be misleading because models that solely incorporate RTs do not appear to be based on the best variable to include in this particular comparison, and if we were to take into account other variables we would see that this percentage is considerably lower. Hence, not all the variables that can be included in the models hold the same level of validity or relevance, indicating the importance of judicious selection among the variables to be considered and manipulated.

In contrast, our results when classifying participants using CL and RT or only CL, as dependent variables, are much more consistent with each other than those using only RT and allow us to make a more appropriate model selection. In the following section, we will justify this statement by means of a simulation study.

### Model simulations

In order to study possible explanations and thus to verify in which situations the comparison between models is more accurate, we have used a simulation approach. Specifically, we generated data following each 2HT and SDT model version (Classic, CL, RT, and CL+RT versions), with parameter values based on each model’s estimations when fitted to data from Juola et al. (2019). We then fitted the various versions of the SDT and 2HT models to the simulated data and performed a model comparison based on AIC. Simulation details and results are described in Appendix E Tables 20, 21, 22 and 23.

Based on the simulations performed, the models that include CLs or CLs and RTs together resulted in selection of the true model for a large majority of subjects. However, when neither of these variables was considered, nearly one-third of the subjects resulted in incorrect model comparisons. Considering the results of the simulations, we can expect that the individual model selections for the experimental data are more reliable when based on CL or CL+RT and, therefore, the individual selections when CL and CL+RT are taken into account should be consistent with each other.

With the simulation results in mind, it is not surprising that of the 47 participants in the Juola et al. (2019) experiment, there are 38 subjects whose data are best fit by a continuous SDT model and 9 by a discrete 2HT model when CLs are analyzed. On the other hand, 41 subjects’ data were best fit by the SDT model and 6 by the 2HT model, leaving only three subjects with inconsistent best-model selections (see Table 4). Furthermore, the results of the simulations indicated that when CLs and RTs were not modeled, the frequency of selection of the true model was lower, so one would expect a decrease in the frequency of selection of the true model in the experimental data leading to the erroneous conclusion that there will be an increased number of subjects who fit the 2HT better than the SDT model when comparing classical models or models that only include RTs. This is precisely what we found when fitting experimental data from Juola et al. (2019).

Thus, based on the simulation results, the usefulness of MPT-DC can be improved with the appropriate selection and inclusion of multiple dependent variables. For example, the conclusions as to whether the recognition model of each participant is better described by a discrete or a continuous model depends on the selection of such variables. Yet why is it that only when CL and RT, or only CL, are included, that the true models are selected as the best ones? One possible reason is that the MPT-DC model takes advantage of information that allows it to differentiate model branches (e.g., CLs are higher in detecting old than in guessing old) thus improving estimates and power. This issue has been analyzed by simulations in the study by Heck et al. (2018b), where it was found that, when the distributions of RTs are equal for the different branches, the model does not benefit from the inclusion of this variable and therefore the estimates will be equivalent to those of a classical MPT model. Another possible explanation is that comparisons between models with RT with two bins are worse than those with CLs with three bins, not due to the amount of information each variable yields but to the number of bins each variable has. Increasing the number of bins can allow us to be more precise about the shape of the variable’s distribution. However, this also means that we must discretize the same set of observed data into more subcategories and, therefore, there is a greater risk of having bins with very few or even no observations. This might affect the estimation capability of the model, and thereby prevent the calculation of standard errors of estimation and the use of chi-squared fit indices. In fact, we have not been able to fit 2HT models or estimate standard errors with three RT bins, since the information matrix was not invertible, something that can occur with insufficient numbers of observations per bin. Unfortunately, despite having a relatively high number of trials per subject (100 trials per tree and subject in our case), we cannot ensure adequate observations solely based on this number. This is because the combination of branches and bins of RTs and CLs can lead to sparse data in certain cells, particularly when dealing with extreme probabilities. Nonetheless, the choice of the number of RT bins will depend, in practice, on the substantive question. If the question of interest lies in a measure of the relative speed of cognitive processes, two RT categories may be sufficient. For example, stating responses of detection states as faster than those of guessing implies that the probability of being fast is greater than that of guessing, which does not necessarily require a three-bin categorization of slow, medium, and fast responses. A simple division into slow and fast may suffice.

## Conclusion

The purpose of the present study is to analyze the advantages of jointly modeling the continuous and discrete variables typically studied in recognition memory paradigms using the 2HT and SDT models. To answer this question, we have explored whether the inclusion of CLs and RTs in MPT-DC models (1) improves the estimation accuracy of classical recognition models, (2) allows us to address new research questions, and (3) encourages us to rethink old ones (i.e., the discrete vs. continuous dilemma).

Concerning the first of these goals, we can conclude that, indeed, the inclusion of variables by means of MPT-DC allows the reduction of standard errors for parameter estimates. However, what may be true for one model may not be true for another. Specifically, we find a reduction in the uncertainty of the estimates when adding CL in the 2HT models, but only when CL and RT are added simultaneously is there an obvious improvement in accuracy in both SDT and 2HT models. It should be mentioned that this result is not necessarily generalizable to other quantitative variables of interest (e.g., eye tracking, mouse trajectory) or to other models (e.g., the two-low-threshold and one-high-threshold model). Therefore, the experimenter wishing to use MPT-DC must rely on data (e.g., comparing models) and theory to choose which variables to include in the MPT-DC model.

As to whether MPT-DC can expand the scope of testable research questions, we have concluded that by jointly modeling CL and RT we can study effects that cannot be studied when analyzing these variables separately. Juola et al. (2019) studied the effects of grouping the data into relative target frequency blocks; confidence level blocks; or RT blocks, but not all three variables at once. The data indicated that, unlike some previous research indicating similar effects of these variables, all three methods of grouping the data yielded differences in the receiver-operating characteristic (ROC) curves. However, it was not possible to estimate the distribution of CLs or RTs associated with each response category or cognitive state, nor to study the interactions between the two variables or how they might interact with relative target frequency. On the contrary, with the 2HT-CL-RT and SDT-CL-RT models, we were able to detect, among others effects, patterns such as (a) “old” responses tend to be of high confidence, and confidence is generally higher for responses emanating from the *detect old* state than from the *guess* states; (b) low-confidence responses are more likely than medium-confidence responses only for the *guess old* state; and (c) the inverse relationship between CLs and RTs is not fulfilled in the *guess old* state in 2HT models nor in the FA categories in SDT models. That said, studying the continuous variables simultaneously as dependent variables allows us to make inferences about the relationships between processes and their outcomes in an integrated manner, avoiding methodological issues derived from including quantitative variables as factors. In addition, although it was not the aim of our study, MPT-DC models can be used to disentangle response strategies or styles (Heck & Erdfelder, 2020). For example, whether extreme styles of confidence ratings tend to result in “high-fast” and “low-fast” responses rather than more typical response styles that result in “high-fast” and “low-slow” responses.

To address the third research question, on whether our modeling proposal allows us to reach new conclusions on old research questions, we faced the continuous-process versus discrete-state recognition model dilemma. Our results allowed us to reconsider several aspects of this matter. First, our results suggest that the question of whether recognition memory is continuous or discrete may need to be rephrased. Do subjects always fit a discrete or a continuous model better? The answer to that question seems to be negative. Our results indicate that only 23 out of 47 subjects always fit the same model, regardless of the variable included. This leads us to the second question. Does the validity of the model comparison depend on measured variables? On one hand, we find that adding more DVs, regardless of the model fitted or the type of DV added, apparently can have a negative impact on the goodness-of-fit of the model. The more DVs we added, the number of subjects’ data for which the goodness-of-fit test was maintained decreased. This seems to indicate that these tests do not tell us much about the fit of each model, but rather that the fit is influenced by the number of categories we are modeling. As mentioned above, by adding more categories, we subdivide the number of trials into more cells, which decreases the number of observations per category and seems to have an effect on the estimate of *χ*^2^ (Davis-Stober, 2009). On the contrary, it is worth noting that our simulation results indicate that only when comparing 2HT and SDT models that include CL and RT simultaneously or CLs alone can we actually identify the model that generates data correctly. The experimental results have also been consistent with the simulations, since if the selections made with CL and CL+RT are correct, as indicated by the simulations, then model selections made with CL or CL+RT should match, which is what we found when using the actual data. In short, according to our results, the comparison between discrete and continuous models depends on the included variables, and we should give more consideration to the model selection made when both CL and RTs or only CLs are taken into account. Third, individual differences impede us from answering the dilemma in a deterministic way. Bearing in mind the relevant variables analyzed in the particular sample used by Juola et al. (2019), there are between 81% and 87% of subjects’ data that fit better to a continuous model, and between 19% and 13% to a discrete one. This could indicate that participants have different response strategies Tourangeau et al. (2000), or that other factors come into play that have not been studied, which make the data fit better to one model or the other, or even that there is a more general or hybrid model, encompassing both the SDT and 2HT models.

The use of MPT-DC models has demonstrated their methodological and theoretical usefulness for modeling the effects of discrete and quantitative variables simultaneously in a recognition memory paradigm. However, as we have discussed above, the experimenter must choose which variables are most relevant to study. The motivation for choosing one or another variable may be substantial to the results of both theoretical and methodological considerations. In this study we have seen that the accuracy of the estimated parameters and model comparisons improve when modeling CLs and CLs with RTs. However, with another sample or other models this conclusion might be different. To construct a protocol on how to select the variables of interest, new methodological studies would have to be carried out (e.g., those that manipulate the number of bins and observations, the distribution of the variable to be included, the cognitive process probabilities). Furthermore, due to the relative recency of the MPT-DC models, several lines of research on the use of these models remain open. Among them we highlight the interest in studying which MPT-DC procedure is optimal for each experimental situation. If the current procedures differ in how they include the data, it seems logical to think that depending on the distribution of the data and the knowledge we have about it, different procedures should apply to different situations. For example, it has been impossible for us to establish three RT bins due to insufficient observations per bin. This problem might be solved by using a parameterized MPT-DC procedure since, unlike the nonparametric one, it uses the data set instead of discretizing it into bins. On the other hand, as we have suggested, due to the interest that MPT-DC has as a method to include variables in SDT models, we would have to modify or create libraries that allow the use of MPT-DC parametric procedures adapted to continuous models, something that to our knowledge is not currently possible. Hence, despite the encouraging results of our study in favor of the use of MPT-DC models, there are still several practical aspects to be developed and investigated regarding their use.

### Alternative modeling approaches

In addition to Heck and Erdfelder’s (2016) proposal to extend the MPT to account for continuous variables using nonparametric methods, there are parametric versions such as those by Klauer and Kellen (2018) and Heck et al. (2018a) that fit fully continuous RT distributions. Klauer and Kellen’s (2018) version is specialized for fitting RTs assumed to follow ex-Gaussian distributions and assumes sequential processes. One of its major advantages is the ability to study the duration of each cognitive process in the sequence. This procedure can be fitted using the “RT-MPT” library (Klauer & Kellen, 2018), although it is designed specifically for RTs. However, it would not be difficult to extend the library to other distributions and thus make it applicable to other continuous variables. Additionally, Klauer and Kellen’s (2018) proposal requires serial processes and does not allow for parallel processes, unlike the proposals of Heck and Erdfelder (2016) and Heck et al. (2018b). In summary, the latter two proposals have the advantage of being applicable to a multitude of continuous variables (RT, eye tracking, cursor movement) and are flexible enough to study a wide variety of models in social psychology, basic psychology, neuroscience, and more, at the cost of being less explicit about the true nature of the processes involved. For example, they estimate the distributions of branches but do not specify the distributional components of the processes involved in them. Furthermore, Starns (2018) proposed a model called the race model, which has a similar structure to an MPT-DC model but integrates RTs by studying the time between the start of a trial and the occurrence of a detection or the participant’s decision to guess. This model does not allow for the study of process durations but rather estimates how long participants take before making a guess; a crucial point for understanding the trade-off between speed and accuracy. An alternative to the race model in the nonparametric MPT-DC framework has been proposed by Heck and Erdfelder (2020).

When it comes to fitting models to a dataset with multiple subjects, we usually must choose between fitting a model to aggregated data from multiple subjects or fitting a model for each individual subject. In this choice, it is often preferred to fit MPT models individually as aggregated data can alter the shape of the distributions we want to study. However, individual fits can be quite poor when there are few observations per subject (Chechile, 2009). As a solution to this dilemma, hierarchical MPT-DC models allow us to estimate distribution shapes quite reliably, even in situations with few observations per subject. However, hierarchical MPT-DC fits have two issues when used to derive individual fits. The first is that they assume that all subjects follow the same model. It is worth noting that our data, supported by simulations, indicates that some subjects fit better to SDT models while others fit better to 2HT models (it is possible that there is a general model that encompasses both SDT and 2HT, which would allow for the application of a single hierarchical model). The second issue is that current hierarchical libraries, for parametric MPT-DC models (“RT-MPT” from Klauer & Kellen, 2018) and nonparametric models (e.g., “TreeBUGS” from Heck, Arnold, et al., 2018a; see also Nestler & Erdfelder, 2023, for a random effects MPT modelling approach.) do not allow fitting of SDT models reparametrized as multinomial models. Therefore, it was not feasible to use hierarchical MPT-DC procedure in the present study.

On the other hand, instead of using MPT-DC models, one can turn to other types of cognitive models that allow for the joint analysis of continuous and discrete variables. Among these, diffusion models stand out as a specific version of evidence-accumulation models that can be used to analyze recognition memory. For example, the circular diffusion model of continuous-outcome source (Zhou et al., 2021) is a circular model, adapted from the diffusion model, applied to a continuous-source memory retrieval task. Unlike existing source retrieval models that attribute all response variability to memory, the circular diffusion model decomposes the noise into variability arising from both memory and decision-making processes. These findings suggest that in the task of continuous-source memory recall, there exists a memory strength threshold that must be reached to retrieve information about the stimulus source. Below this threshold, no information is retrieved, leading to guessing responses. Furthermore, the study suggests that participants’ confidence is controlled in an old/new recognition task, ruling out the possibility that participants’ guesses are simply due to a lack of recognition of the items. Additionally, we have the models proposed by Donkin et al. (2013), that combine a discrete state representation with a particular sequential sampling model called the linear ballistic accumulator (LBA; Brown & Heathcote, 2008). This model has an accumulator for each available response, and the response is triggered when the amount of evidence in one of the accumulators reaches a specific criterion. The LBA model uses evidence accumulators for each of the possible responses in the task (e.g., “change” or “no change”). The model assumes that some trials are “guessing” trials, where the average accumulation rate is the same for both accumulators, and the remaining trials are “memory” trials, where accumulation rates are determined by the presented stimulus. In other words, the distribution of accumulation rates across trials is a discrete mixture of various types of trials.

Similarly, attempts have been made to model continuous variables such as RTs using SDT models. According to strength theory, both RTs and CLs can be defined as a function of distance to a criterion. For example, an exponential decay function was used to fit the distribution of RTs as a function of distance to criteria (Atkinson & Juola, 1973). However, this approach did not produce a significant improvement in the model fit to justify the additional parameters. It is crucial to note that these functions usually assume an inverse relationship between CL and RT. However, if this assumption is not entirely true, it could lead to incorrect predictions. To address this problem, MPT-DC models estimate RTs distributions within each branch, which allows us to examine whether the inverse relationship between RTs and CLs holds across branches. In our research, we have found that this is not the case. This knowledge of the relationship between RT and CL is valuable, as it helps to avoid potentially erroneous predictions based on nondirectly observable assumptions that do not hold when fitted to the real data.

In conclusion, depending on our theoretical models (e.g., models of continuous processes versus models of discrete processes), the type of theoretical hypotheses we want to test as researchers (e.g., hypotheses regarding fast/slow responses versus expectations of changes in the parameters of continuous RT distributions), and the distribution of our data, it may be better to employ one approach over the other. However, it would be interesting to leverage the strengths of each modeling approach to address the same scientific objective, thereby gaining robustness in our conclusions through converging evidence. By combining these approaches, we can enhance our understanding and provide more comprehensive insights into the cognitive processes under investigation.

## Data Availability

Datasets from Juola et al. (2019) are available in https://osf.io/y78mk/ while simulated data and other material are available in https://osf.io/ezb3g/.

## Appendix A. Model formulation

In this article we have shown both 2HT and SDT models as MPTs, as well as several examples in which a branch of the model can be extended, by means of an MPT-DC, to integrate CLs and/or RTs. In MPTs, the sequences of processes or nodes form the branches of the tree model. To obtain the probability of a given branch, we must multiply the probabilities of the nodes that belong to that branch. Likewise, the probability of the observed responses is the result of adding the probabilities of those branches that give rise to the same response. In the following, we show the equations that allow modeling the observed responses of all the models developed in this study.

It should be mentioned that, following Province and Rouder (2012), CLs and RTs distributions are conditionally independent, which means that the distribution of the variables associated with a state will be identical regardless of how the state was reached. This leads to the following assumptions:

1. If there are two paths (i.e., MPT branches) to reach the same state, the distribution of CLs and RTs is the same for all existing paths. Therefore, the CL and RT distributions associated with the *guess old* states will be identical for the old or the new stimulus and the same applies to the confidence distribution associated with the *guess new* states.
2. The CL and RT distributions associated with a cognitive state do not change by an increase or decrease in the probability of entering that state. Therefore, changes in the probability of guessing states resulting from manipulation of the proportion of targets presented will not affect the CLs of the *guess old* and *guess new* states.

Table 6

**Table 6:** Two-high-threshold model

Stimulus	2HT	Category
Old	*do* + (1 − *do*) · *g*_*j*_	Hit
	(1 − *do*) · (1 − *g*_*j*_)	Miss
New	(1 − *dn*) · *g*_*j*_	FA
	*dn* + (1 − *dn*) · (1 − *g*_*j*_)	CR

Table 7

**Table 7:** Confidence levels for the two-high-threshold model

Stimulus	2HT-CL	Category	CL
Old	*do* · *Ldo*_1_ + (1 − *do*) · *g*_*j*_ · *Lgo*_1_	Hit	high
	*do* · (1 − *Ldo*_1_) · *Ldo*_2_ + (1 − *do*) · *g*_*j*_ · (1 − *Lgo*_1_) · *Lgo*_2_	Hit	medium
	*do* · (1 − *Ldo*_1_) · (1 − *Ldo*_2_) + (1 − *do*) · *g*_*j*_ · (1 − *Lgo*_1_) · (1 − *Lgo*_2_)	Hit	low
	(1 − *do*) · (1 − *g*_*j*_) · *Lgn*_1_	Miss	high
	(1 − *do*) · (1 − *g*_*j*_) · (1 − *Lgn*_1_) · *Lgn*_2_	Miss	medium
	(1 − *do*) · (1 − *g*_*j*_) · (1 − *Lgn*_1_) · (1 − *Lgn*_2_)	Miss	low
New	(1 − *dn*) · *g*_*j*_ · *Lgo*_1_	FA	high
	(1 − *dn*) · *g*_*j*_ · (1 − *Lgo*_1_) · *Lgo*_2_	FA	medium
	(1 − *dn*) · *g*_*j*_ · (1 − *Lgo*_1_) · (1 − *Lgo*_2_)	FA	low
	*dn* · *Ldn*_1_ + (1 − *dn*) · (1 − *g*_*j*_) · *Lgn*_1_	CR	high
	*dn* · (1 − *Ldn*_1_) · *Ldn*_2_ + (1 − *dn*) · (1 − *g*_*j*_) · (1 − *Lgn*_1_) · *Lgn*_2_	CR	medium
	*dn* · (1 − *Ldn*_1_) · (1 − *Ldn*_2_) + (1 − *dn*) · (1 − *g*_*j*_) · (1 − *Lgn*_1_) · (1 − *Lgn*_2_)	CR	low

Table 8

**Table 8:** Reaction times for the two-high-threshold model

Stimulus	2HT-RT	Category	RT
Old	*do* · *Hdo*_1_ + (1 − *do*) · *g*_*j*_ · *Hgo*_1_	Hit	fast
	*do* · (1 − *Hdo*_1_) + (1 − *do*) · *g*_*j*_ · (1 − *Hgo*_1_)	Hit	slow
	(1 − *do*) · (1 − *g*_*j*_) · *Hgn*_1_	Miss	fast
	(1 − *do*) · (1 − *g*_*j*_) · (1 − *Hgn*_1_)	Miss	slow
New	(1 − *dn*) · *g*_*j*_ · *Hgo*_1_	FA	fast
	(1 − *dn*) · *g*_*j*_ · (1 − *Hgo*_1_)	FA	slow
	*dn* · *Hdn*_1_ + (1 − *dn*) · (1 − *g*_*j*_) · *Hgn*_1_	CR	fast
	*dn* · (1 − *Hdn*_1_) + (1 − *dn*) · (1 − *g*_*j*_) · (1 − *Hgn*_1_)	CR	slow

Table 9

**Table 9:** Confidence levels and reaction times for the two-high-threshold model

Stimulus	2HT-CL-RT	Category	CL	RT
Old	*do* · *Ldo*_1_ · *Hdo*_1_ + (1 − *do*) · *g*_*j*_ · *Lgo*_1_ · *Hgo*_1_	Hit	high	fast
	*do* · *Ldo*_1_ · (1 − *Hdo*_1_) + (1 − *do*) · *g*_*j*_ · *Lgo*_1_ · (1 − *Hgo*_1_)	Hit	high	slow
	*do* · (1 − *Ldo*_1_) · *Ldo*_2_ · *Hdo*_2_ + (1 − *do*) · *g*_*j*_ · (1 − *Lgo*_1_) · *Lgo*_2_ · *Hgo*_2_	Hit	medium	fast
	*do* · (1 − *Ldo*_1_) · *Ldo*_2_ · (1 − *Hdo*_2_) + (1 − *do*) · *g*_*j*_ · (1 − *Lgo*_1_) · *Lgo*_2_ · (1 − *Hgo*_2_)	Hit	medium	slow
	*do* · (1 − *Ldo*_1_) · (1 − *Ldo*_2_) · *Hdo*_3_ + (1 − *do*) · *g*_*j*_ · (1 − *Lgo*_1_) · (1 − *Lgo*_2_) · *Hgo*_3_	Hit	low	fast
	*do* · (1 − *Ldo*_1_) · (1 − *Ldo*_2_) · (1 − *Hdo*_3_) + (1 − *do*) · *g*_*j*_ · (1 − *Lgo*_1_) · (1 − *Lgo*_2_) · (1 − *Hgo*_3_)	Hit	low	slow
	(1 − *do*) · (1 − *g*_*j*_) · *Lgn*_1_ · *Hgn*_1_	Miss	high	fast
	(1 − *do*) · (1 − *g*_*j*_) · *Lgn*_1_ · (1 − *Hgn*_1_)	Miss	high	slow
	(1 − *do*) · (1 − *g*_*j*_) · (1 − *Lgn*_1_) · *Lgn*_2_ · *Hgn*_2_	Miss	medium	fast
	(1 − *do*) · (1 − *g*_*j*_) · (1 − *Lgn*_1_) · *Lgn*_2_ · (1 − *Hgn*_2_)	Miss	medium	slow
	(1 − *do*) · (1 − *g*_*j*_) · (1 − *Lgn*_1_) · (1 − *Lgn*_2_) · *Hgn*_3_	Miss	low	fast
	(1 − *do*) · (1 − *g*_*j*_) · (1 − *Lgn*_1_) · (1 − *Lgn*_2_) · (1 − *Hgn*_3_)	Miss	low	slow
New	(1 − *dn*) · *g*_*j*_ · *Lgo*_1_ · *Hgo*_1_	FA	high	fast
	(1 − *dn*) · *g*_*j*_ · *Lgo*_1_ · (1 − *Hgo*_1_)	FA	high	slow
	(1 − *dn*) · *g*_*j*_ · (1 − *Lgo*_1_) · *Lgo*2 · *Hgo*_2_	FA	medium	fast
	(1 − *dn*) · *g*_*j*_ · (1 − *Lgo*_1_) · *Lgo*2 · (1 − *Hgo*_2_)	FA	medium	slow
	(1 − *dn*) · *g*_*j*_ · (1 − *Lgo*_1_) · (1 − *Lgo*2) · *Hgo*_3_	FA	low	fast
	(1 − *dn*) · *g*_*j*_ · (1 − *Lgo*_1_) · (1 − *Lgo*2) · (1 − *Hgo*_3_)	FA	low	slow
	*dn* · *Ldn*_1_ · *Hdn*_1_ + (1 − *dn*) · (1 − *g*_*j*_) · *Lgn*_1_ · *Hgn*_1_	CR	high	fast
	*dn* · *Ldn*_1_ · (1 − *Hdn*_1_) + (1 − *dn*) · (1 − *g*_*j*_) · *Lgn*_1_ · (1 − *Hgn*_1_)	CR	high	slow
	*dn* · (1 − *Ldn*_1_) · *Ldn*_2_ · *Hdn*_2_ + (1 − *dn*) · (1 − *g*_*j*_) · (1 − *Lgn*_1_) · *Lgn*_2_ · *Hgn*_2_	CR	medium	fast
	*dn* · (1 − *Ldn*_1_) · *Ldn*_2_ · (1 − *Hdn*_2_) + (1 − *dn*) · (1 − *g*_*j*_) · (1 − *Lgn*_1_) · *Lgn*_2_ · (1 − *Hgn*_2_)	CR	medium	slow
	*dn* · (1 − *Ldn*_1_) · (1 − *Ldn*_2_) · *Hdn*_3_ + (1 − *dn*) · (1 − *g*_*j*_) · (1 − *Lgn*_1_) · (1 − *Lgn*_2_) · *Hgn*_3_	CR	low	fast
	*dn* · (1 − *Ldn*_1_) · (1 − *Ldn*_2_) · (1 − *Hdn*_3_) + (1 − *dn*) · (1 − *g*_*j*_) · (1 − *Lgn*_1_) · (1 − *Lgn*_2_) · (1 − *Hgn*_3_)	CR	low	slow

Table 10

**Table 10:** Signal detection theory model

Stimulus	SDT	Category
Old	$1-\upphi \left(\frac{c_j-{d}^{\prime }}{\sigma}\right)$	Hit
	$\upphi \left(\frac{c_j-{d}^{\prime }}{\sigma}\right)$	Miss
New	1 − ϕ(*c*_*j*_)	FA
	ϕ(*c*_*j*_)	CR

Table 11

**Table 11:** Reaction times for the signal detection theory model

Stimulus	SDT-RT	Category	RT
Old	$1-\upphi \left(\frac{c_k-{d}^{\prime }}{\sigma}\right)\cdot Hs{o}_1$	Hit	fast
	$1-\upphi \left(\frac{c_k-{d}^{\prime }}{\sigma}\right)\cdot \left(1- Hs{o}_1\right)$	Hit	slow
	$\upphi \left(\frac{c_k-{d}^{\prime }}{\sigma}\right)\cdot Hs{n}_1$	Miss	fast
	$\upphi \left(\frac{c_k-{d}^{\prime }}{\sigma}\right)\cdot \left(1- Hs{n}_1\right)$	Miss	slow
New	[1 − ϕ(*c*_*k*_)] · *Hro*_1_	FA	fast
	[1 − ϕ(*c*_*k*_)] · (1 − *Hro*_1_)	FA	slow
	ϕ(*c*_*k*_) · *Hrn*_1_	CR	fast
	ϕ(*c*_*k*_) · (1 − *Hrn*_1_)	CR	slow

Table 12

**Table 12:** Confidence levels for the signal detection theory model

Stimulus	SDT-CL	Category	CL
Old	$1-\upphi \left(\frac{\Delta {c}_5+\Delta {c}_4+\Delta {c}_3+\Delta {c}_2+\Delta {c}_{1j}-{d}^{\prime }}{\sigma}\right)$	Hit	high
	$\upphi \left(\frac{\Delta {c}_5+\Delta {c}_4+\Delta {c}_3+\Delta {c}_2+\Delta {c}_{1j}-{d}^{\prime }}{\sigma}\right)-\upphi \left(\frac{\Delta {c}_4+\Delta {c}_3+\Delta {c}_2+\Delta {c}_{1j}-{d}^{\prime }}{\sigma}\right)$	Hit	medium
	$\upphi \left(\frac{\Delta {c}_4+\Delta {c}_3+\Delta {c}_2+\Delta {c}_{1j}-{d}^{\prime }}{\sigma}\right)-\upphi \left(\frac{\Delta {c}_3+\Delta {c}_2+\Delta {c}_{1j}-{d}^{\prime }}{\sigma}\right)$	Hit	low
	$\upphi \left(\frac{\Delta {c}_{1j}-{d}^{\prime }}{\sigma}\right)$	Miss	high
	$\upphi \left(\frac{\Delta {c}_2+\Delta {c}_{1j}-{d}^{\prime }}{\sigma}\right)-\upphi \left(\frac{\Delta {c}_{1j}-{d}^{\prime }}{\sigma}\right)$	Miss	medium
	$\upphi \left(\frac{\Delta {c}_3+\Delta {c}_2+\Delta {c}_{1j}-{d}^{\prime }}{\sigma}\right)-\upphi \left(\frac{\Delta {c}_2+\Delta {c}_{1j}-{d}^{\prime }}{\sigma}\right)$	Miss	low
New	1 − ϕ(∆*c*_5_ + ∆*c*_4_ + ∆*c*_3_ + ∆*c*_2_ + ∆*c*_1*j*_)	FA	high
	ϕ(∆*c*_5_ + ∆*c*_4_ + ∆*c*_3_ + ∆*c*_2_ + ∆*c*_1*j*_) − ϕ(∆*c*_4_ + ∆*c*_3_ + ∆*c*_2_ + ∆*c*_1*j*_)	FA	medium
	ϕ(∆*c*_4_ + ∆*c*_3_ + ∆*c*_2_ + ∆*c*_1*j*_) − ϕ(∆*c*_3_ + ∆*c*_2_ + ∆*c*_1*j*_)	FA	low
	ϕ(∆*c*_1*j*_)	CR	high
	ϕ(∆*c*_2_ + ∆*c*_1*j*_) − ϕ(∆*c*_1*j*_)	CR	medium
	ϕ(∆*c*_3_ + ∆*c*_2_ + ∆*c*_1*j*_) − ϕ(∆*c*_2_ + ∆*c*_1*j*_)	CR	low

Table 13

**Table 13:** Confidence Levels and Reaction Times for the Signal Detection Theory Model.

Stimulus	SDT-CL-RT	Category	CL	RT
Old	$\left[1-\upphi \left(\frac{\Delta {c}_5+\Delta {c}_4+\Delta {c}_3+\Delta {c}_2+\Delta {c}_{1j}-{d}^{\prime }}{\sigma}\right)\right]\cdot Hs{o}_1$	Hit	high	fast
	$\left[1-\upphi \left(\frac{\Delta {c}_5+\Delta {c}_4+\Delta {c}_3+\Delta {c}_2+\Delta {c}_{1j}-{d}^{\prime }}{\sigma}\right)\right]\cdot \left(1- Hs{o}_1\right)$	Hit	medium	slow
	$\left[\upphi \left(\frac{\Delta {c}_5+\Delta {c}_4+\Delta {c}_3+\Delta {c}_2+\Delta {c}_{1j}-{d}^{\prime }}{\sigma}\right)-\upphi \left(\frac{\Delta {c}_4+\Delta {c}_3+\Delta {c}_2+\Delta {c}_{1j}-{d}^{\prime }}{\sigma}\right)\right]\cdot \left( Hs{o}_2\right)$	Hit	low	fast
	$\left[\upphi \left(\frac{\Delta {c}_5+\Delta {c}_4+\Delta {c}_3+\Delta {c}_2+\Delta {c}_{1j}-{d}^{\prime }}{\sigma}\right)-\upphi \left(\frac{\Delta {c}_4+\Delta {c}_3+\Delta {c}_2+\Delta {c}_{1j}-{d}^{\prime }}{\sigma}\right)\right]\cdot \left(1- Hs{o}_2\right)$	Hit	high	slow
	$\left[\upphi \left(\frac{\Delta {c}_4+\Delta {c}_3+\Delta {c}_2+\Delta {c}_{1j}-{d}^{\prime }}{\sigma}\right)-\upphi \left(\frac{\Delta {c}_3+\Delta {c}_2+\Delta {c}_{1j}-{d}^{\prime }}{\sigma}\right)\right]\cdot \left( Hs{o}_3\right)$	Hit	medium	fast
	$\left[\upphi \left(\frac{\Delta {c}_4+\Delta {c}_3+\Delta {c}_2+\Delta {c}_{1j}-{d}^{\prime }}{\sigma}\right)-\upphi \left(\frac{\Delta {c}_3+\Delta {c}_2+\Delta {c}_{1j}-{d}^{\prime }}{\sigma}\right)\right]\cdot \left( Hs{o}_3\right)$	Hit	low	slow
	$\upphi \left(\frac{\Delta {c}_{1j}-{d}^{\prime }}{\sigma}\right)\cdot \left( Hs{n}_1\right)$	Miss	high	fast
	$\upphi \left(\frac{\Delta {c}_{1j}-{d}^{\prime }}{\sigma}\right)\cdot \left(1- Hs{n}_1\right)$	Miss	medium	slow
	$\left[\upphi \left(\frac{\Delta {c}_2+\Delta {c}_{1j}-{d}^{\prime }}{\sigma}\right)-\upphi \left(\frac{\Delta {c}_{1j}-{d}^{\prime }}{\sigma}\right)\right]\cdot \left( Hs{n}_2\right)$	Miss	low	fast
	$\left[\upphi \left(\frac{\Delta {c}_2+\Delta {c}_{1j}-{d}^{\prime }}{\sigma}\right)-\upphi \left(\frac{\Delta {c}_{1j}-{d}^{\prime }}{\sigma}\right)\right]\cdot \left(1- Hs{n}_2\right)$	Miss	high	slow
	$\left[\upphi \left(\frac{\Delta {c}_3+\Delta {c}_2+\Delta {c}_{1j}-{d}^{\prime }}{\sigma}\right)-\upphi \left(\frac{\Delta {c}_2+\Delta {c}_{1j}-{d}^{\prime }}{\sigma}\right)\right]\cdot \left( Hs{n}_3\right)$	Miss	medium	fast
	$\left[\upphi \left(\frac{\Delta {c}_3+\Delta {c}_2+\Delta {c}_{1j}-{d}^{\prime }}{\sigma}\right)-\upphi \left(\frac{\Delta {c}_2+\Delta {c}_{1j}-{d}^{\prime }}{\sigma}\right)\right]\cdot \left(1- Hs{n}_3\right)$	Miss	low	slow
New	[1 − ϕ(∆*c*_5_ + ∆*c*_4_ + ∆*c*_3_ + ∆*c*_2_ + ∆*c*_1*j*_)] · *Hro*_1_	FA	high	fast
	[1 − ϕ(∆*c*_5_ + ∆*c*_4_ + ∆*c*_3_ + ∆*c*_2_ + ∆*c*_1*j*_)] · (1 − *Hro*_1_)	FA	high	slow
	[ϕ(∆*c*_5_ + ∆*c*_4_ + ∆*c*_3_ + ∆*c*_2_ + ∆*c*_1*j*_) − ϕ(∆*c*_4_ + ∆*c*_3_ + ∆*c*_2_ + ∆*c*_1*j*_)] · (*Hro*_2_)	FA	medium	fast
	[ϕ(∆*c*_5_ + ∆*c*_4_ + ∆*c*_3_ + ∆*c*_2_ + ∆*c*_1*j*_) − ϕ(∆*c*_4_ + ∆*c*_3_ + ∆*c*_2_ + ∆*c*_1*j*_)] · (1 − *Hro*_2_)	FA	medium	slow
	[ϕ(∆*c*_4_ + ∆*c*_3_ + ∆*c*_2_ + ∆*c*_1*j*_) − ϕ(∆*c*_3_ + ∆*c*_2_ + ∆*c*_1*j*_)] · (*Hro*_3_)	FA	low	fast
	[ϕ(∆*c*_4_ + ∆*c*_3_ + ∆*c*_2_ + ∆*c*_1*j*_) − ϕ(∆*c*_3_ + ∆*c*_2_ + ∆*c*_1*j*_)] · (1 − *Hro*_3_)	FA	low	slow
	ϕ(∆*c*_1*j*_) · (*Hrn*_1_)	CR	high	fast
	ϕ(∆*c*_1*j*_) · (1 − *Hrn*_1_)	CR	high	slow
	[ϕ(∆*c*_2_ + ∆*c*_1*j*_) − ϕ(∆*c*_1*j*_)] · (*Hrn*_2_)	CR	medium	fast
	[ϕ(∆*c*_2_ + ∆*c*_1*j*_) − ϕ(∆*c*_1*j*_)] · (1 − *Hrn*_2_)	CR	medium	slow
	[ϕ(∆*c*_3_ + ∆*c*_2_ + ∆*c*_1*j*_) − ϕ(∆*c*_2_ + ∆*c*_1*j*_)] · (*Hrn*_3_)	CR	low	fast
	[ϕ(∆*c*_3_ + ∆*c*_2_ + ∆*c*_1*j*_) − ϕ(∆*c*_2_ + ∆*c*_1*j*_)] · (1 − *Hrn*_3_)	CR	low	slow

## Appendix B. Description analysis for aggregated data

Table 14

**Table 14:** Frequency by category and confidence

Target Ratio	Hit			Miss			FA			CR		
	high	medium	low	high	medium	low	high	medium	low	high	medium	low
35%	1802	389	78	250	434	102	320	206	62	517	453	87
50%	1210	277	48	324	408	83	383	219	79	828	723	118
65%	768	195	28	250	341	63	326	268	79	1174	1050	158

*Note*. The frequency for the all subjects, *N* = 47, of each category and confidence-rating, is shown for each target proportion condition (35% to 65%).

Table 15

**Table 15:** RT sample means

Target Ratio		Response-Confidence Level					
		new-high	new-medium	new-low	old-low	old-medium	old-high
35%	success	1250.9	1494.0	1847.1	1767.7	1537.1	1021.5
		(585.0)	(778.5)	(1085.0)	(676.2)	(817.0)	(1100.0)
	failure	1254.9	1511.8	1901.7	1518.8	1549.7	1105.2
		(808.3)	(1026.0)	(1229.6)	(913.8)	(920.7)	(1042.7)
50%	success	1215.3	1421.7	1944.3	1958.0	1531.6	1100.9
		(660.8)	(876.6)	(1073.6)	(650.8)	(922.8)	(1026.3)
	failure	1207.5	1397.7	1736.0	1765.0	1584.9	1336.2
		(1346.0)	(844.4)	(1030.4)	(733.8)	(940.6)	(936.4)
65%	success	1187.6	1405.7	2036.5	2126.4	1699.1	1195.5
		(713.7)	(1008.1)	(2017.3)	(958.8)	(841.6)	(1497.6)
	failure	1186.2	1314.9	1791.1	2219.4	1812.0	1376.6
		(1485.8)	(1162.8)	(3129.7)	(734.9)	(970.1)	(1025.4)

*Note*. Aggregate mean RT (ms) and standard deviation (in parentheses) across all subjects, *N* = 47, are shown for each response-confidence combination (new-high/new-medium/new-old/new-low/old-low/old/medium/old-high) and for each target proportion condition (35% to 65%). Whether the outcome of the response was correct or wrong is also specified (success/failure).

## Appendix C. Model predictions

**Analysis**

Statistical comparisons for each model on individual parameter estimates are shown below. The $\hat{g}$and $\hat{c}$, respectively, were analyzed with repeated-measures univariate ANOVAs. Since Mauchly’s sphericity test was rejected, the reported *F*s will always refer to Greenhouse–Geisser corrections. Three additional analyses were performed by means of a mixed general linear model with subjects as the random factor. To choose the variance and covariance matrix for each mixed model, we followed the principle of parsimony. Using the *G*^2^−test, we searched for the matrix with the minimum number of parameters that did not cause a significant loss of fit with respect to the saturated model (unstructured matrix). The probability of each CL-bin was analyzed by means of a mixed general linear model with subjects as the random factor, with two fixed within-participant factors, establishing a diagonal variance-covariance matrix (*G*^2^= 317.4, *df*=66, *p* = .999). The within-participant factors were the *states* (detect new/detect old/guess new/guess old) and *confidence-levels* (high/medium/low). The probability of fast RT bins was also analyzed by means of a mixed general linear model with participants as the random factor, *states* (detect new/detect old/guess new/guess old), and *confidence-levels* (high/medium/low) as within-participant factors. A compound symmetry variance-covariance matrix was selected (*G*^2^=208.7, *df*=76, *p* = .999). Both models included the interaction with *states* × *confidence-levels*. For the probability of *fast* bins in the SDT model, the within-participant factors were the *response-categories* (CR/FA/Miss/Hit) and *confidence-levels* (high/medium/low), establishing a symmetry variance-covariance matrix (*G*^2^=212.1, *df*=76, *p* = .999). Partial eta squared (*η*_*p*_^2^) was used as a measure of effect size. For all significant effects, post-hoc pairwise comparisons Tukey’s Test with Bonferroni correction was performed. The significance level (alpha) was set to .05.

## Results

Regarding the effect of relative target frequency blocks in the 2HT-CL-RT model, it is observed that $\hat{g}$ (see Fig. 13) is different across the relative target frequency blocks, *F*(2, 84) = 18.55, *p* < .001, *η*_*p*_^2^ = .287, and that the relationship between this variables is positive and linear *F*(1, 46) = 28.86, *p* < .001, *η*_*p*_^2^ = .386.

The results of the *states* × *confidence-levels* analysis (see Fig. 14) shows a rejection of the null hypothesis *F*(2, 287) = 386.8, *p* < .001, *η*_*p*_^2^ = .729. In particular, the hypothesis of probability equality of the different CLs is rejected in the states of *detect new F*(2, 83) = 56.55, *p* < .001, *η*_*p*_^2^ = .576; *detect old*, *F*(2, 77) = 928, *p* < .001, *η*_*p*_^2^ = .96; *guess new* states, *F*(2, 84) = 69.00, *p* < .001, *η*_*p*_^2^ = .621; and *guess old* states, *F*(2, 85) = 86.34, *p* < .001, *η*_*p*_^2^ = .586. Responses are more likely to be high confidence than medium confidence (*p* < .001) for all comparisons except *guess new* states, for which *p* = .158, and medium-confidence responses are more likely than low-confidence ones (*p* < .001 for all above comparisons).

Regarding the RT bins (see Fig. 15), the hypothesis that short RTs probabilities are the same at all three CLs was rejected, *F*(2, 506) = 23.39, *p* < .001, *η*_*p*_^2^ = .085, with a higher probability of *short* RTs in high-confidence responses than in medium (*p* < .001) or low confidence (*p* < .001) and no evidence of differences between medium and low confidence (*p* = .999). However, in *guess old* states*, F*(2, 506) = 11.51, *p* < .001, *η*_*p*_^2^ = .044,there are more rapid responses at low CLs than at medium levels (*p* = .014). Likewise, the speed difference between high and low-confidence responses is significantly larger in *detect old* than in *guess new* (*p* = .02) or *guess old* (*p* = .006) states, something that does not hold true for the *detect new* states (*p* = .6 for *guess old* and *p* = .53 for *guess new* comparisons)*.* In short, there is an inverse linear relationship between CLs and RTs in *detect new, F*(1, 46) = 71.08, *p* < .001, *η*_*p*_^2^ = .607, *detect old*, *F*(1, 46) = 1661.3, *p* < .001, *η*_*p*_^2^ = .973, and *guess new*, *F*(1, 46) = 41.23, *p* < .001, *η*_*p*_^2^ = .473,state.

Considering the SDT-CL-RT model, (see Figure 16), results shows that *c*_*kj*_ varies between the *J* = 3 relative target frequency blocks, *F*(2, 80) = 22.83, *p* < .001, *η*_*p*_^2^ = .332, where the relationship between the proportion of targets and the estimated criterion is linear and negative, *F*(1, 46) = 38.23, *p* < .001, *η*_*p*_^2^ = .454 (see dotted line in Fig. 16). In addition, inside each target proportion condition, the criteria for different CLs increase linearly from *c*_1*j*_ to *c*_5*j*_, *F*(1, 46) = 164.3, *p* < .001, *η*_*p*_^2^ = .781.

Concerning the probability of *short* RT in the SDT model (see Fig. 17), there is a significant effect of the *confidence-levels* factor, *F*(2, 506) = 60.7, *p* < .001, *η*_*p*_^2^ = .193, but not of the *response-category* factor, *F*(3, 506) = 2.23, *p* = .084, *η*_*p*_ = .013. In the first analysis, responses for high are faster than those for medium (*p* < .001) and low CLs (*p* < .001). The *response-category* × *confidence*-*level* interaction effect is also significant, *F*(6,506) = 15.69, *p* < 0.001, *η*_*p*_^2^ = .15. Specifically, the probability of the RTs being short is higher at high CLs versus medium and low, for the Hit (*p* < .001), Miss (*p* = .004 and *p* = .009 for medium and low CLs comparisons, respectively) and CR (*p* = .006 and *p* < .001, for medium and low CLs comparisons, respectively), but not for the FA category. In FAs we do not find speed differences between medium and low CLs (*p* = .136).

## Appendix D. Goodness-of-fit χ^2^ bootstrap test and *AICc*

Table 16

**Table 16:** Goodness-of-fit and AIC for classic models

	2HT			SDT		
*Id*	*χ* ^*2*^ *(df = 1)*	Critical Value	*AIC*_*C*_	*χ* ^*2*^ *(df = 1)*	Critical Value	*AIC*_*C*_
1	0.041	4.39	10.04	0.049	5.266	10.05
2	1.14	3.51	11.14	0.564	3.444	10.56
3	1.44	5.211	11.44	1.482	3.997	11.48
4	0.191	2.829	10.19	0.143	3.66	10.14
5	0.048	3.219	10.05	0.01	2.406	10.01
6	0.029	3.172	10.03	0.002	4.254	10
7	0.765	4.78	10.76	0.83	2.905	10.83
8	0.737	3.132	10.74	0.603	2.109	10.6
9	0.119	4.679	10.12	0	3.145	10
10	0.595	4.1	10.59	0.497	5.735	10.5
11	0.015	3.93	10.02	0.004	2.628	10
12	2.271	5.149	12.27	0.018	4.412	10.02
13	0.148	3.259	10.15	0.221	3.775	10.22
14	0.066	3.953	10.07	0.068	3.437	10.07
15	0.08	4.249	10.08	0.073	1.909	10.07
16	1.423	5.86	11.42	0.261	4.193	10.26
17	3.609	4.243	13.61	2.37	4.749	12.37
18	0.742	5.581	10.74	0.724	3.885	10.72
19	0.568	4.59	10.57	0.739	3.288	10.74
20	2.014	3.845	12.01	1.883	2.964	11.88
21	0.882	3.733	10.88	0.909	2.23	10.91
22	4.902	4.454	14.9	4.026	4.961	14.03
23	0.167	3.566	10.17	0.022	3.234	10.02
24	4.021	4.24	14.02	3.291	3.974	13.29
25	0.837	5.05	10.84	0	2.529	10
26	4.334	3.375	14.33	4.595	3.095	14.59
27	0.123	4.805	10.12	0.081	3.896	10.08
28	1.32	3.509	11.32	1.024	4.65	11.02
29	0.141	4.583	10.14	0.132	1.891	10.13
30	0.214	4.915	10.21	0.294	2.874	10.29
31	0.221	3.97	10.22	0.02	3.603	10.02
32	0.987	5.152	10.99	0.845	2.566	10.84
33	0.095	5.222	10.1	0.004	4.074	10
34	1.393	3.735	11.39	1.293	2.882	11.29
35	0.518	4.261	10.52	0.496	2.316	10.5
36	0.761	2.934	10.76	0.655	2.972	10.65
37	0.232	5.316	10.23	0.232	3.992	10.23
38	0.751	5.254	10.75	0.847	2.171	10.85
39	3.1	5.268	13.1	0.671	5.199	10.67
40	0.013	4.01	10.01	0.295	4.953	10.29
41	0.036	3.608	10.04	0.004	2.599	10
42	1.209	4.891	11.21	1.469	4.224	11.47
43	3.679	5.394	13.68	3.517	2.977	13.52
44	0.299	4.314	10.3	0.008	4.232	10.01
45	0.812	6.679	10.81	0.846	3.877	10.85
46	0.042	4.239	10.04	0.035	6.256	10.03
47	0.875	4.399	10.88	0.244	3.952	10.24

Table 17

**Table 17:** Goodness-of-fit & AIC for CL models

	2HT-CL			SDT-CL		
*Id*	*χ* ^*2*^ *(df=17)*	Critical Value	*AIC*_*C*_	*χ* ^*2*^ *(df = 21)*	Critical Value	*AIC*_*C*_
1	27.004	28.346	53	35.511	32.817	53.51
2	16.885	29.262	42.89	28.111	36.486	46.11
3	8.306	19.213	34.31	9.811	23.591	27.81
4	22.306	29.269	48.31	21.433	32.347	39.43
5	33.537	20.485	59.54	23.126	24.173	41.13
6	33.953	27.94	59.95	17.124	33.166	35.12
7	32.77	22.016	58.77	22.612	33.259	40.61
8	14.166	23.581	40.17	22.378	29.381	40.38
9	34.745	28.712	60.75	27.726	33.6	45.73
10	12.67	18.155	38.67	14.145	20.356	32.15
11	67.691	32.494	93.69	52.038	39.118	70.04
12	32.4	31.703	58.4	48.925	30.053	66.93
13	52.241	25.569	78.24	57.715	28.392	75.72
14	15.4	24.455	41.4	20.813	28.589	38.81
15	17.521	34.85	43.52	14.71	33.198	32.71
16	46.845	28.69	72.85	44.849	32.034	62.85
17	41.479	27.803	67.48	30.295	38.175	48.29
18	27.693	30.509	53.69	35.263	38.949	53.26
19	12.366	30.029	38.37	11.832	35.259	29.83
20	30.864	27.011	56.86	32.431	26.554	50.43
21	19.208	31.832	45.21	22.077	32.8	40.08
22	79.367	26.336	105.37	87.963	32.788	105.96
23	34.301	19.363	60.3	33.259	23.207	51.26
24	24.158	18.735	50.16	34.105	22.699	52.11
25	34.88	30.878	60.88	40.875	34.376	58.88
26	37.122	24.854	63.12	42.531	29.189	60.53
27	36.331	33.504	62.33	47.468	31.64	65.47
28	71.929	31.509	97.93	85.479	39.434	103.48
29	19.029	33.128	45.03	23.03	35.307	41.03
30	9.101	30.816	35.1	11.033	36.056	29.03
31	32.142	27.707	58.14	42.415	34.841	60.41
32	25.936	29.323	51.94	28.541	38.562	46.54
33	13.841	23.903	39.84	16.719	27.854	34.72
34	22.779	30.956	48.78	30.144	31.719	48.14
35	41.754	22.469	67.75	47.91	24.393	65.91
36	33.227	27.404	59.23	39.097	29.929	57.1
37	46.629	27.287	72.63	33.465	30.287	51.47
38	29.015	32.073	55.02	34.473	28.572	52.47
39	44.705	30.298	70.71	47.485	30.416	65.48
40	5.787	8.926	31.79	7.775	10.983	25.78
41	25.618	29.304	51.62	27.555	30.822	45.55
42	28.858	30.551	54.86	22.089	30.987	40.09
43	31.839	21.228	57.84	34.385	22.351	52.39
44	22.941	28.765	48.94	23.2	34.766	41.2
45	21.797	31.225	47.8	28.926	36.494	46.93
46	35.722	34.445	61.72	21.955	33.406	39.95
47	104.663	23.639	130.66	104.792	23.45	122.79

Table 18

**Table 18:** Goodness-of-fit & AIC for RT models

	2HT-RT	SDT-RT				
*Id*	*χ* ^*2*^ *(df = 9)*	Critical Value	*AIC*_*C*_	*χ* ^*2*^ *(df = 9)*	Critical Value	*AIC*_*C*_
1	14.574	16.769	32.57	15.88	16.712	33.88
2	22.933	14.783	40.93	24.664	17.239	42.66
3	16.188	17.472	34.19	14.981	15.387	32.98
4	8.243	17.977	26.24	8.247	19.986	26.25
5	41.081	16.002	59.08	38.013	14.291	56.01
6	24.328	18.591	42.33	25.058	14.92	43.06
7	11.009	19.446	29.01	10.74	17.71	28.74
8	11.879	14.953	29.88	12.167	18.179	30.17
9	13.083	20.701	31.08	10.745	20.545	28.74
10	7.778	15.461	25.78	6.752	15.171	24.75
11	12.681	17.996	30.68	12.641	18.071	30.64
12	28.7	18.75	46.7	27.949	16.89	45.95
13	9.488	18.673	27.49	9.711	17.911	27.71
14	19.475	18.823	37.47	19.013	17.947	37.01
15	4.006	19.538	22.01	3.67	19.102	21.67
16	56.581	20.214	74.58	55.282	15.447	73.28
17	45.822	20.433	63.82	44.979	18.346	62.98
18	20.612	17.784	38.61	20.45	15.449	38.45
19	20.444	21.606	38.44	19.294	19.002	37.29
20	18.903	13.815	36.9	19.281	17.266	37.28
21	14.84	16.428	32.84	14.283	18.753	32.28
22	74.113	16.928	92.11	72.145	19.266	90.14
23	38.14	16.188	56.14	37.44	16.198	55.44
24	13.215	19.566	31.21	12.929	18.742	30.93
25	23.655	19.602	41.65	23.389	19.28	41.39
26	41.851	15.941	59.85	42.03	16.829	60.03
27	12.849	20.451	30.85	13.307	15.765	31.31
28	43.51	17.806	61.51	48.242	15.732	66.24
29	29.198	16.805	47.2	28.944	16.626	46.94
30	29.645	16.321	47.64	28.182	16.555	46.18
31	45.641	17.744	63.64	52.084	16.23	70.08
32	12.886	18.56	30.89	12.46	14.884	30.46
33	24.733	16.552	42.73	22.906	15.932	40.91
34	6.956	18.796	24.96	5.927	17.362	23.93
35	5.021	16.699	23.02	5.184	15.667	23.18
36	10.458	23.566	28.46	9.784	16.482	27.78
37	22.554	18.825	40.55	21.604	17.904	39.6
38	5.587	21.116	23.59	4.851	16.514	22.85
39	15.264	20.514	33.26	12.795	21.019	30.79
40	31.669	15.955	49.67	34.855	17.107	52.85
41	5.5	19.525	23.5	5.414	21.64	23.41
42	29.736	16.147	47.74	30.213	16.073	48.21
43	27.996	15.764	46	26.389	18.382	44.39
44	8.863	16.547	26.86	8.286	17.875	26.29
45	7.59	14.817	25.59	7.333	16.586	25.33
46	25.092	19.494	43.09	22.175	17.287	40.17
47	38.208	17.827	56.21	37.038	18.623	55.04

Table 19

**Table 19:** Goodness-of-fit & AIC for CL+RT models

	2HT-CL-RT			SDT-CL-RT		
*Id*	*χ* ^*2*^ *(df = 41)*	Critical Value	*AIC*_*C*_	*χ* ^*2*^ *(df = 45)*	Critical Value	*AIC*_*C*_
1	45.245	52.604	95.25	51.314	57.225	93.31
2	51.183	51.727	101.18	62.686	56.899	104.69
3	40.375	42.294	90.38	40.708	44.148	82.71
4	33.624	49.393	83.62	32.584	51.181	74.58
5	81.282	42.74	131.28	69.009	44.061	111.01
6	67.057	52.638	117.06	49.999	50.439	92
7	51.352	53.784	101.35	38.796	52.841	80.8
8	37.325	45.773	87.32	44.822	50.65	86.82
9	65.901	61.419	115.9	60.202	60.133	102.2
10	29.05	37.274	79.05	30.267	37.031	72.27
11	105.045	53.784	155.04	89.643	54.954	131.64
12	65.837	46.417	115.84	85.199	54.841	127.2
13	78.106	49.809	128.11	83.771	54.607	125.77
14	37.827	43.954	87.83	42.475	57.01	84.47
15	46.278	56.378	96.28	41.578	61.856	83.58
16	116.582	48.011	166.58	108.769	52.094	150.77
17	109.748	65.408	159.75	99.077	64.897	141.08
18	68.329	60.74	118.33	75.686	62.027	117.69
19	52.318	70.697	102.32	49.706	67.955	91.71
20	51.208	41.142	101.21	54.426	45.037	96.43
21	48.382	66.251	98.38	51.171	62.326	93.17
22	130.831	48.137	180.83	137.604	50.658	179.6
23	73.171	33.979	123.17	71.981	36.667	113.98
24	43.367	40.688	93.37	52.751	47.849	94.75
25	82.571	58.365	132.57	89.118	62.917	131.12
26	75.445	50.38	125.45	80.688	45.687	122.69
27	61.31	61.888	111.31	71.165	64.577	113.17
28	126.757	58.324	176.76	138.514	59.647	180.51
29	65.774	67.466	115.77	64.486	58.068	106.49
30	60.358	63.645	110.36	58.322	61.862	100.32
31	87.442	51.261	137.44	101.879	51.89	143.88
32	61.594	67.303	111.59	60.978	64.801	102.98
33	39.875	42.263	89.88	39.606	45.279	81.61
34	42.727	51.925	92.73	49.838	52.99	91.84
35	55.057	39.366	105.06	59.539	41.557	101.54
36	53.859	49.516	103.86	57.036	53.518	99.04
37	82.433	54.103	132.43	64.72	50.667	106.72
38	62.926	62.854	112.93	67.32	66.263	109.32
39	68.612	53.15	118.61	67.998	54.935	110
40	36.028	22.686	86.03	40.548	26.342	82.55
41	47.78	57.133	97.78	47.039	52.523	89.04
42	82.753	55.159	132.75	71.691	47.541	113.69
43	57.046	38.499	107.05	57.126	41.048	99.13
44	61.276	64.828	111.28	61.294	66.495	103.29
45	59.939	60.101	109.94	60.368	66.955	102.37
46	76.106	60.959	126.11	60.78	63.189	102.78
47	135.158	41.21	185.16	135.394	48.086	177.39

## Appendix E. Simulation details and results

To disentangle model selection patterns in real data, we performed a model comparison analysis for simulated data. For 2,000 replicates, data were generated for each version of the 2HT and SDT model (Classic, CL, RT, and CL+RT versions) with parameter values based on each model estimates when fitted to data from Juola et al. (2019). The 2HT (Table 20) and SDT (Table 21) estimated parameters for the aggregate data of the 47 subjects that are shown below.

To test the impact of the variables included in the estimation models on the correct selection of discrete or continuous models, 2HT and SDT models that include CLs and RTs, only one of these variables or even none of them, were fitted to both 2HT and SDT simulated data. Model selection was also based on AIC. The simulation and model fitting were performed in R with the MPTinR library. Simulation data and code are available at https://osf.io/ezb3g.

Table 22 shows the frequency and percentage of discrete and continuous model selections as best fit models to the simulated data that follows the 2HT model. Including CLs results in a true model selection of 81.8% while only including RTs or no other variables reduces that percentage to 65.2 and 67.6, respectively. More remarkably, 86.0% of the best fit models are correct when both CLs and RTs are included. Thus, there is a considerably higher percentage of correct model selections when CLs or CLs and RTs are included.

When the data-generating model is the SDT model (Table 23), the classical SDT and 2HT models are selected as the best model in 66.1% and 33.9% of the cases, respectively. As for the models incorporating RT, we find similar percentages, namely 66.5% and 33.5% for the SDT and 2HT models, respectively. This shows that, although there is a higher percentage of correct selections, there is still about 1 out of 3 model comparisons that produce incorrect selections. But again, we find that when CLs or both CLs and RTs are taken into account, the percentages of correct selections are much higher (96.2% and 97.2%, respectively).

Table 20

**Table 20:** Aggregate 2HT estimates

	*dn*	*do*	*g*_*1*_	*g*_*2*_	*g*_*3*_															
2HT	.39	.37	.36	.46	0.59															
2HT-RT	.34	.41	.34	.43	0.55															
2HT-CL	.26	.46	.29	.38	0.49															
2HT-CL-RT	.23	.48	.29	.37	0.48															
	*Hdn*_*1*_	*Hdn*_*2*_	*Hdn*_*3*_	*Hdo*_*1*_	*Hdo*_*2*_	*Hdo*_*3*_	*Hgn*_*1*_	*Hgn*_*2*_	*Hgn*_*3*_	*Hgo*_*1*_	*Hgo*_*2*_	*Hgo*_*3*_	*Ldn*_*1*_	*Ldn*_*2*_	*Ldo*_*1*_	*Ldo*_*2*_	*Lgn*_*1*_	*Lgn*_*2*_	*Lgo*_*1*_	*Lgo*_*2*_
2HT																				
2HT-RT	.55			.74			.52			.52										
2HT-CL													.72	.97	.90	.1	.37	.83	.54	.77
2HT-CL-RT	.58	.53	. 52	.75	.45	.00	.59	.50	.34	.64	. 38	.38	.76	1	.89	.97	.37	. 83	.54	.77

*Note*. The classical parameters of the 2HT model are found in the first table. In the bottom table, the *H* and *L* parameters are those associated with RTs and CLs, respectively.

Table 21

**Table 21:** Aggregate SDT estimates

	*d′*	*σ*	*c*_*31*_	*c*_*32*_	*c*_*33*_	*Δc*_*2*_	*Δc*_*3*_	*Δc*_*4*_	*Δc*_*5*_	*Hrn*_*1*_	*Hrn*_*2*_	*Hrn*_*3*_	*Hro*_*1*_	*Hro*_*2*_	*Hro*_*3*_	*Hsn*_*1*_	*Hsn*_*2*_	*Hsn*_*3*_	*Hso*_*1*_	*Hso*_*2*_	*Hso*_*3*_
SDT	1	1.02	.35	.58	.76																
SDT-RT	1	1.02	.35	.58	.76					.54			.51			.51			.66		
	*d′*	*σ*	*c*_*11*_	*c*_*12*_	*c*_*13*_	*Δc*_*2*_	*Δc*_*3*_	*Δc*_*4*_	*Δc*_*5*_	*Hrn*_*1*_	*Hrn*_*2*_	*Hrn*_*3*_	*Hro*_*1*_	*Hro*_*2*_	*Hro*_*3*_	*Hsn*_*1*_	*Hsn*_*2*_	*Hsn*_*3*_	*Hso*_*1*_	*Hso*_*2*_	*Hso*_*3*_
SDT-CL	1.11	1.28	−.59	−.38	−.24	.82	.14	.09	.39												
SDT-CL-RT	1.11	1.28	−.59	−.38	−.24	.82	.14	.09	.39	.59	.51	.33	.63	.37	.41	.59	.49	.36	.32	.41	.31

*Note*. The classical parameters of the SDT model are found in the first two columns. The *c*_*1*_*-c*_*5*_ estimates represent the criteria changes through target proportion manipulations. When CL-bins are ignored (SDT and SDT-RT models) these decision criteria parameters separate “new” and “old” responses. When CL-bins are considered, this represents the *c*_*1j*_ criterion (that separates “high-new” from “medium-new” responses). To obtain criteria values for other confidence-response bins *∆c* estimates must be added following equations (1) to (4). The *H* parameters are those associated with the RT bins.

Table 22

**Table 22:** Model selection for 2HT simulated data

Model		n.par	AIC.best	%AIC.best
Classic	SDT	5	650	32.5
	2HT	5	1352	67.6
CL	SDT	9	363	18.15
	2HT	13	1637	81.85
RT	SDT	9	697	34.85
	2HT	9	1303	65.15
CL-RT	SDT	21	281	14.05
	2HT	25	1719	85.95

*Note.* SDT vs. 2HT model selection for data generated by the 2HT model simulated data is presented for classic (Classic rows), confidence levels (CL rows), reaction time (RT row) confidence level and reaction time (CL-RT row) model versions. The frequency and percentage of each model (SDT vs. 2HT) selection appears in the AIC.best columns.

Table 23

**Table 23:** Model selection for SDT simulated data

**Model**		**n.par**	**AIC.best**	**%AIC.best**
Classic	SDT	5	1322	66.1
	2HT	5	678	33.9
CL	SDT	9	1925	96.25
	2HT	13	75	3.75
RT	SDT	9	1330	66.5
	2HT	9	670	33.5
CL-RT	SDT	21	1944	97.2
	2HT	25	56	2.8

*Note.* SDT vs. 2HT model selection for data generated by the SDT model simulated data are presented for classic (Classic rows), confidence levels (CL rows), reaction time (RT row) confidence level and reaction time (CL-RT row) model versions. The frequency and percentage of each model (SDT vs. 2HT) selection appears in the AIC.best columns.

## Footnotes

1 The model is presented in a binary version, where each of the three CL-BIN (High/Medium/Low) bin probabilities (*L*_1_^′^, *L*_2_^′^ and *L*_3_^′^, respectively) is formulated as: *L*_1_^′^ = *L*_1_; *L*_2_^′^ = (1 − *L*_1_)*L*_2_;and *L*_3_^′^ = (1 − *L*_1_)(1 − *L*_2_).
